# Parkinson’s disease neurons exhibit alterations in mitochondrial quality control proteins

**DOI:** 10.1038/s41531-023-00564-3

**Published:** 2023-08-08

**Authors:** Chun Chen, David McDonald, Alasdair Blain, Emily Mossman, Kiera Atkin, Michael F. Marusich, Roderick Capaldi, Laura Bone, Anna Smith, Andrew Filby, Daniel Erskine, Oliver Russell, Gavin Hudson, Amy E. Vincent, Amy K. Reeve

**Affiliations:** 1 Wellcome Centre for Mitochondrial Research, Translational and Clinical Research Institute, Newcastle University, Newcastle upon Tyne, UK; 2 Innovation, Methodology and Application Research Theme, Biosciences Institute, Newcastle University, Newcastle upon Tyne, UK; 3 Flow Cytometry Core Facility, Newcastle University, Newcastle upon Tyne, UK; 4 mAbDx, Inc., Eugene, OR USA; 5 Cellstate Biosciences, Tucson, AZ USA; 6 Wellcome Centre for Mitochondrial Research, Biosciences Institute, Newcastle University, Newcastle upon Tyne, UK

**Keywords:** Parkinson's disease, Cellular neuroscience

## Abstract

Mitochondrial dysfunction has been suggested to contribute to Parkinson’s disease pathogenesis, though an understanding of the extent or exact mechanism of this contribution remains elusive. This has been complicated by challenging nature of pathway-based analysis and an inability simultaneously study multiple related proteins within human brain tissue. We used imaging mass cytometry (IMC) to overcome these challenges, measuring multiple protein targets, whilst retaining the spatial relationship between targets in post-mortem midbrain sections. We used IMC to simultaneously interrogate subunits of the mitochondrial oxidative phosphorylation complexes, and several key signalling pathways important for mitochondrial homoeostasis, in a large cohort of PD patient and control cases. We revealed a generalised and synergistic reduction in mitochondrial quality control proteins in dopaminergic neurons from Parkinson’s patients. Further, protein-protein abundance relationships appeared significantly different between PD and disease control tissue. Our data showed a significant reduction in the abundance of PINK1, Parkin and phosphorylated ubiquitin^Ser65^, integral to the mitophagy machinery; two mitochondrial chaperones, HSP60 and PHB1; and regulators of mitochondrial protein synthesis and the unfolded protein response, SIRT3 and TFAM. Further, SIRT3 and PINK1 did not show an adaptive response to an ATP synthase defect in the Parkinson’s neurons. We also observed intraneuronal aggregates of phosphorylated ubiquitin^Ser65^, alongside increased abundance of mitochondrial proteases, LONP1 and HTRA2, within the Parkinson’s neurons with Lewy body pathology, compared to those without. Taken together, these findings suggest an inability to turnover mitochondria and maintain mitochondrial proteostasis in Parkinson’s neurons. This may exacerbate the impact of oxidative phosphorylation defects and ageing related oxidative stress, leading to neuronal degeneration. Our data also suggest that that Lewy pathology may affect mitochondrial quality control regulation through the disturbance of mitophagy and intramitochondrial proteostasis.

## Introduction

Mitochondrial dysfunction has long been considered an important pathogenic contributor to Parkinson’s disease (PD)^1,2^. The detrimental effect of dysfunctional mitochondrial oxidative phosphorylation (OxPhos) on the survival of dopaminergic (DA) neurons has been supported by the establishment of experimental PD models using complex I toxins^3–5^. These models manifested with severe substantia nigra (SN) DA neurodegeneration and a response to dopamine supplements.

Loss of complex I function in mice has been shown to cause a loss of neuronal phenotype and Levodopa-responsive parkinsonism, in the absence of overt neurodegeneration^6^. However, despite this, the simultaneous study of SN neurons from individuals with PD and mitochondrial disease revealed differential survival of these neurons between disorders^7,8^. In patients who carry pathogenic maternally inherited mitochondrial DNA (mtDNA) mutations, such as the *m.3243A*>*G* mutation in MELAS (mitochondrial encephalomyopathy, lactic acidosis and stroke-like episodes) or a single, large-scale mtDNA deletion, SN neurons may be affected by comparable levels of OxPhos dysfunction to those seen in PD, however the neuronal population is often maintained^7^. Whilst in individuals with mutations in the *POLG* gene leading to impaired mtDNA polymerisation activity, severe OxPhos deficiency caused primarily by multiple mtDNA deletions acquired over time as in PD and with advancing age, is often associated with neurodegeneration. These somatic mtDNA mutations contribute to declines in complex I and IV activity in PD, POLG and aged SN neurons, however catastrophic neuronal loss is only typically observed in PD and a small number of POLG cases^9,10^. It is well-established that individual tissues exhibit different thresholds of OxPhos dysfunction at which tissue function is compromised^11,12^. This raises the questions whether SN neurons might differentially respond to this dysfunction depending on the condition and underlying mutation, an important consideration given the reliance of these neurons on the function of this organelle. Understanding the mechanism which underlies any SN neuronal adaption will extend our current aetiologic understanding of idiopathic PD and reveal therapeutic targets to achieve protection of these neurons and treatment for PD.

**Fig. 1:**
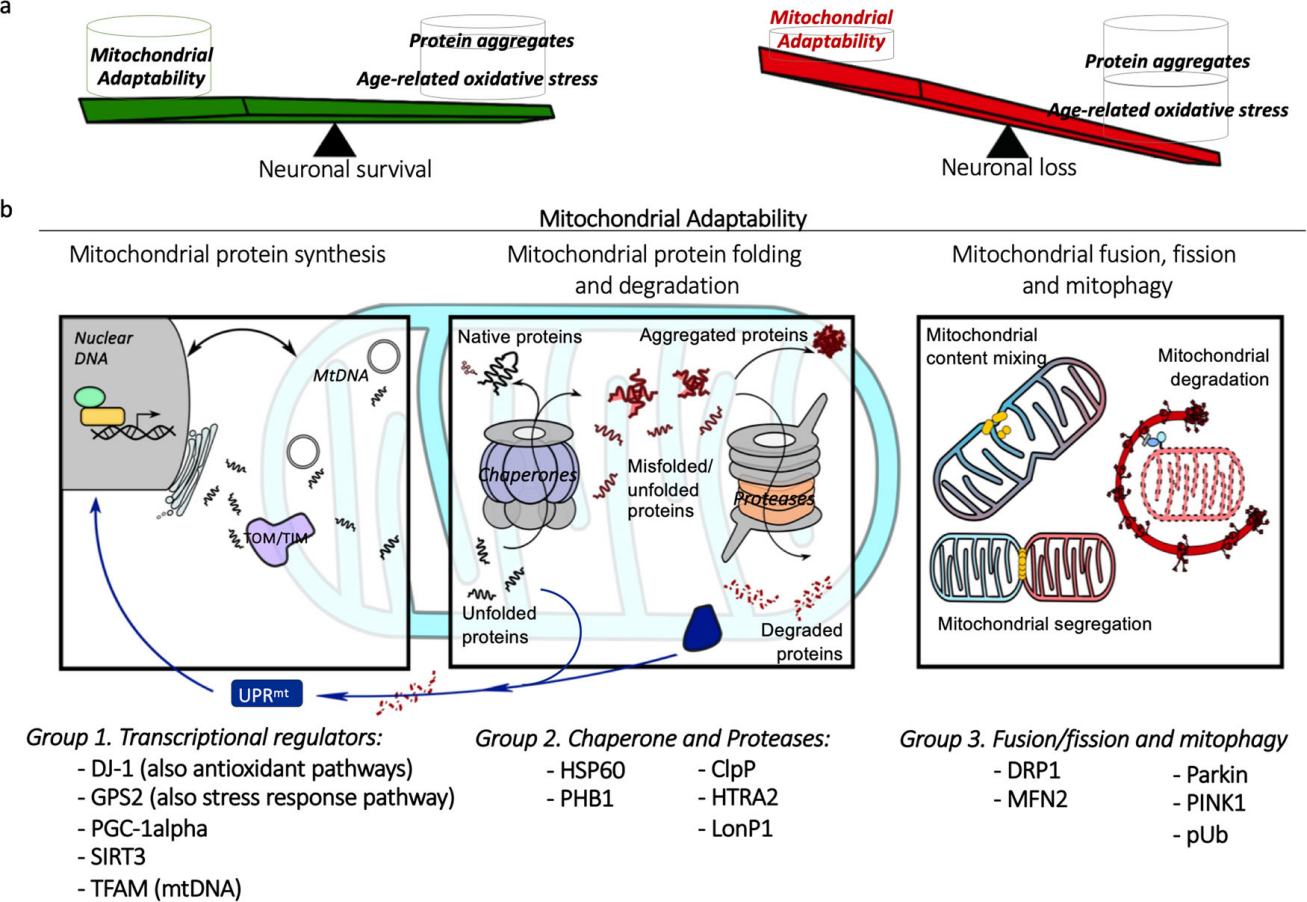
Mitochondrial adaptability in Parkinson’s neurons. Mitochondrial quality control (MQC) operates through the intricate cooperation of three processes: synthesis and import of new building blocks for mitochondria; correct protein folding and degradation to maintain protein homoeostasis within the organelle; alongside mitochondrial dynamics and mitophagy to turnover dysfunctional mitochondria. This MQC system allows neuronal mitochondria to monitor and adapt themselves to a certain degree of damage, such as OxPhos defects, oxidative stress and protein aggregation, without causing interference to neuron homoeostasis. It is hypothesised that the MQC system is impaired in PD; the weakening mitochondrial adaptability is unable to compensate for the increasing burden of oxidative damage and protein degradation that advancing with age, leads to premature neuronal death (**a**). In this study, we investigated the expression change of key MQC signalling proteins on PD neurons using imaging mass cytometry (IMC) (**b**). These tested proteins were categorised into three major groups based on their most well-known function for easy interpretation.

Mitochondria have a surveillance system to adapt to damage and monitor proteostasis and integrity, known as the mitochondrial quality control (MQC) system. This is implemented through intricate signalling pathways between the nucleus and mitochondria^13,14^. It involves collaboration and coordination between mitochondrial protein synthesis and mitochondrial chaperone and protease (such as AAA proteases LonP1 and ClpP) activity, alongside the ubiquitin-proteasome system (UPS) and mitochondrial unfolded protein response (UPR^mt^). This regulates correct intraorganellar protein import, folding and turnover of misfolded proteins, while mitochondrial fusion and fission act to dilute damaged contents between mitochondria, with mitophagy facilitating the clearance of dysfunctional mitochondria (Fig. 1). Since mtDNA encodes only 13 OxPhos subunits^15^, maintenance of mitochondrial proteostasis relies heavily on nuclear and mitochondrial interaction, facilitated by the UPR^mt^ signalling. MtDNA mutations and OxPhos complex deficiency, both features of PD neurons, could cause perturbations to mitochondrial proteostasis via increased ROS generation, interruption to ATP synthesis and membrane potential that impact the import of nuclear-encoded mitochondrial proteins^16,17^. Thus, it is tempting to speculate that such alterations would have a negative effect on the MQC system, given its reliance on nuclear-mitochondrial signalling.

Alpha-synuclein (α-Syn) aggregates, have been shown to hinder the import of mitochondrial proteins through their interaction with mitochondrial outer membrane protein TOM20, resulting in the senescence of mitochondria within DA neurons^18^. Overexpression of α-Syn has been suggested to enhance and prolong UPR^mt^ activity, consequently inducing non-apoptotic neuronal death (necroptosis)^19^. Conversely, manipulation of expression and import of mitochondrial proteases HTRA2 and LONP1 can induce α-Syn aggregation^20^. TRAP1 (tumour necrosis factor type 1 receptor associated protein, also known as HSP75) regulates HTRA2 and PINK1 activity, TRAP1 variants associated with loss-of function are more common in late-onset PD than controls^21^. Furthermore, a study in a small PD cohort found pathogenic mutations affect the proteolytic activity of HTRA2 which serves as a key protease in the UPR^mt^ pathway^22^. We therefore, hypothesised that the functional adaptability of mitochondria to maintain proteostasis and integrity may be compromised by Lewy pathology, leading to accelerated decline of mitochondrial function and increased susceptibility to aged-related OxPhos defects and pathogenic protein aggregation in PD (Fig. 1).

Our previous findings using post-mortem tissues have suggested the occurrence of perturbations in mitochondrial proteostasis within SN neurons of PD cases, showing deficiency in the expression of enzymes involving all five OxPhos complexes, although the mitochondrial population in these neurons is maintained^23^. The low incidence, yet generalised pattern of OxPhos deficiency, alongside a significant decrease of TFAM abundance in PD neurons was not present in neurons with inherited mitochondrial defects or those undergoing normal ageing^8,23^. TFAM, a nuclear-encoded mtDNA transcription factor, is turned over by LonP1 in the mitochondrial matrix and is a downstream target in the nuclear transcriptional cascade regulated by PGC-1α. Importantly, mitochondrial protein synthesis, including of nuclear-encoded OxPhos enzymes that are PGC-1α responsive, has been shown to be down-regulated in PD neurons^24^. These findings are also consistent with single-neuron transcriptomic analysis, which revealed PD-specific impaired expression of OxPhos genes from both genomes, alongside genes involved in mitochondrial biogenesis. Such changes were associated only with disease and not with ageing effects^25^.

To gain a more complete understanding of changes associated with mitochondrial proteostasis and their impact in PD, here we measure a group of signalling factors that are involved in several aspects of the MQC regulatory machinery (Fig. 1). We used imaging mass cytometry on post-mortem fixed midbrain sections. The simultaneous measurement of protein abundance at the single-neuron level allowed assessment of the relationship between signalling factors and their response to OxPhos deficiency, which could provide further insight into changes in pathways and the interactions between them.

## Results

**Fig. 2:**
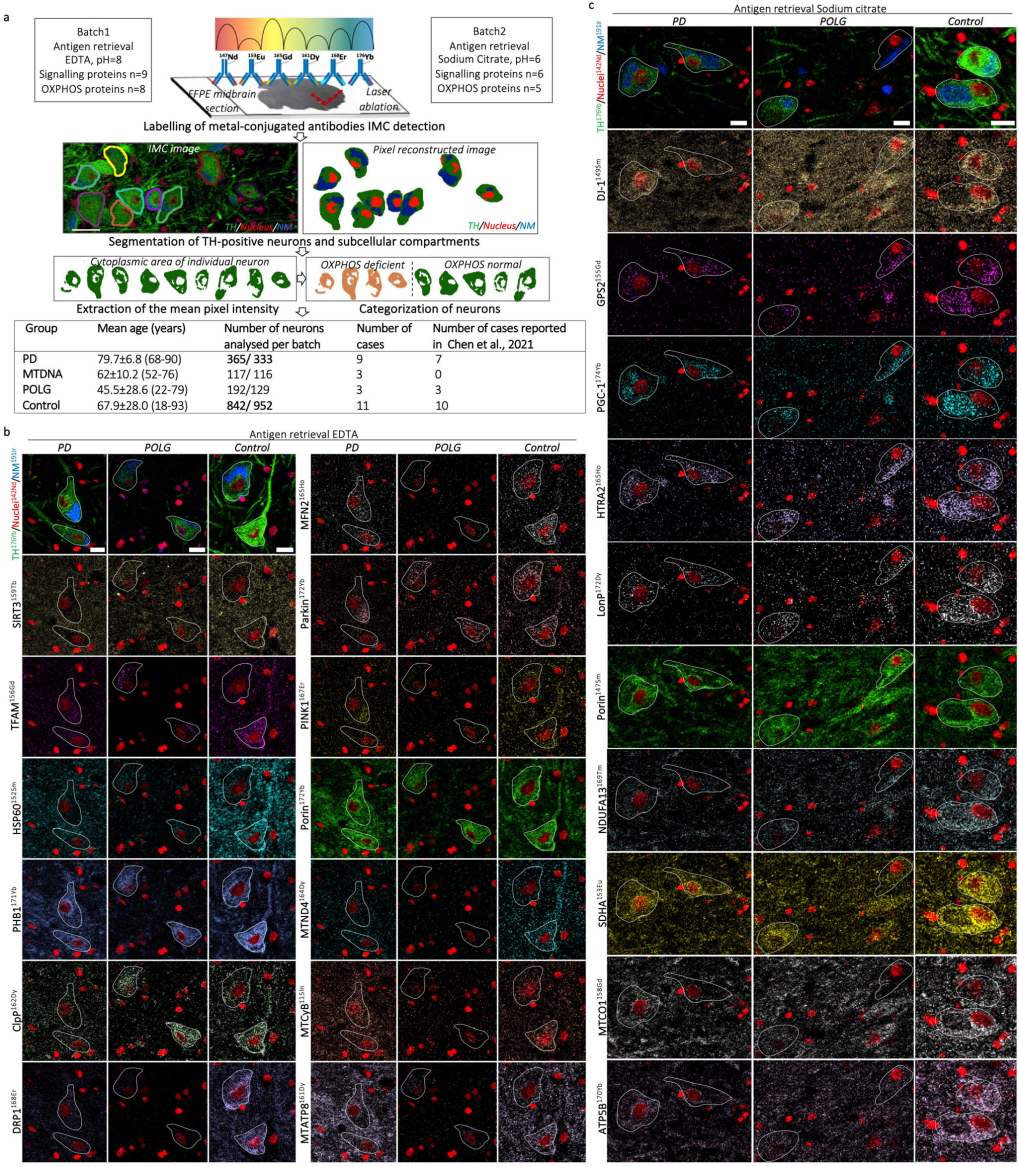
IMC analysis of mitochondrial MQC proteins expression in human SN neurons. **a** An outline of the IMC workflow on FFPE midbrain sections. Immunostaining of metal conjugated antibodies (Table 1) for IMC detection were performed in two experiments, using EDTA (pH = 8) and sodium citrate (pH = 6) buffer respectively for heat-mediated antigen retrieval. Individual dopaminergic neurons within the SN were segmented based on positive tyrosine hydroxylase (TH) staining, a clear nucleus (anti-Histone H3) and intracellular neuromelanin signals (visualised via Ir-intercalator binding); single pixel intensities were extracted, allowing measurement of the mean pixel intensity of each cytoplasmic area for statistical analysis. These neurons were further categorised into those with complex I/IV/V deficiency and those with normal protein levels respectively; the level of OxPhos protein deficiency was statistically defined based on the lower 10% limit of the prediction interval of the control dataset^23^. Differences of the signalling protein expression between the two categories (deficient/normal) of neurons were further analysed to understand their changes in association with OxPhos deficiency. Detailed information of the tissue cohort included in the study were summarised in Supplementary Table 1. Scale bar, 20 µm. Example IMC images were selected from PD08 (male, 90 yrs), POLG01 (female, 90 yrs) and CON04 (female, 54 yrs) (**b**); PD01(male, 70 yrs), POLG02 (male, 59 yrs) and CON09 (male, 88 yrs) (**c**), demonstrating differential signal intensities from test proteins between neurons within selected individuals, and between groups. Scale bar, 20 µm.

Our previous IMC study revealed generalised deficiency involving all five OxPhos complexes in SN neurons of individuals with PD^23^. However, the mechanism that underlies such deficiency remains elusive. To address this gap in our knowledge of the pathogenicity of PD, we expanded the IMC antibody panel to include 15 signalling proteins. These signalling proteins are key participants in the mitochondrial protein synthesis and MQC regulatory pathways (Fig. 1) allowing comprehensive protein profiling. This gives the ability to understand changes in mitochondrial proteostasis in PD. Depending on the optimal heat-induced antigen retrieval buffer, IMC detection of these targeted proteins was conducted in two experiments (EDTA and Sodium Citrate, Fig. 2a). Images of PD, POLG and control neurons demonstrate examples of variable signal within each individual and between groups, for both experiments (Fig. 2b, c). Such variations might be attributed to the varying mtDNA heteroplasmy, the investigated sub-region within the SN, or the differential implication of mitochondrial pathology in the disease.

### Deficiency of MQC proteins in the DA neurons of PD

**Fig. 3:**
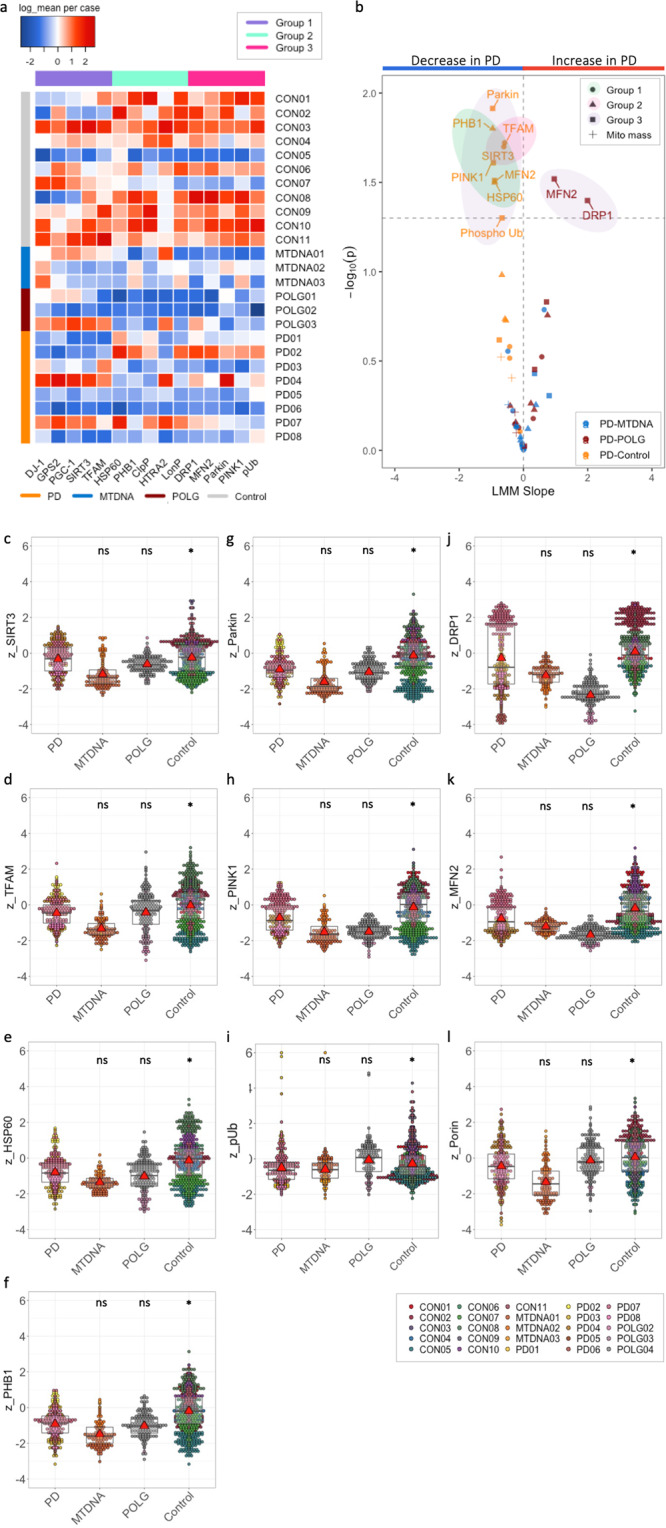
Profiling of MQC protein abundance in disease and control cases. **a** Heatmap demonstration of the average levels of signalling protein abundance for individual cases in groups, ranked in diseases (Supplementary Table 1) and protein groups (Fig. 1b). The mean log-transformed intensity data for each tested protein were normalised across tested individuals (scaled by column). **b**–**l** Using the linear mixed regression modelling (LMM)^26^, difference in the abundance signalling proteins between PD neurons and neurons from mitochondrial disease and healthy controls were analysed. The slopes and *p* values for each tested target from the LMM analysis were summarised into a volcano plot for an integral visualisation (**b**), with the detailed comparison of proteins that showed a significant change, along with the mitochondrial mass marker (Porin/VDAC1) demonstrated (LMM; *p*, *≤0.05; ns > 0.05) (**c**–**l**). Mitochondrial cases with mtDNA point mutation and POLG mutations as disease controls, alongside healthy aged controls were compared to PD group. Each data point represents each paired group (**b**) or single neuron (**c**–**l**); Boxplots show median and mean (triangle) 25th and 75th percentile, with whiskers indicating 95^th^ upper/lower interquartile range.

The single-neuron resolution and quantitative nature of IMC allows measurement of the single pixel intensity for each target protein per cytoplasmic area within each individual DA neuron of the SN. Proteins that regulate mitochondrial fusion and fission (DRP1 and MFN1), mitophagy (PINK1, Parkin and phosphorylated ubiquitin^Ser65^ (pUb^Ser65^)); mitochondrial chaperones (HSP60 and PHB1), mitochondrial proteases (HTRA2 and LonP1), alongside transcription factors (GPS2 and TFAM) showed a high protein level in 10/11 controls, with a trend for decreased levels among most PD and mitochondrial disease cases (Fig. 3a). However, there is also considerable heterogeneity within each group and between targeted proteins (Fig. 3a), for example, PD04 and 07 (Braak stage IV) showed higher levels of 10/15 signalling proteins compared to the remaining PD cases; generalised low levels of signalling protein abundance were also present in one of our healthy control cases (CON05, female, 59 years).

Next, protein levels between PD cases and controls were compared at the single-neuron level. We combined all of the data, using LME modelling^26^ to account for the different number of neurons analysed per individual in each group (Supplementary Table 1). Significant decreases in the abundance of 8/15 signalling proteins were characterised in PD neurons, when compared to healthy controls (Fig. 3b–k). The largest decrease was observed in PHB1 abundance (LME, slope = −0.95, *p* = 0.02), followed by PINK1 (slope = −0.93, *p* = 0.02), MFN2 (slope = −0.90, *p* = 0.03) and HSP60 (slope = −0.89, *p* = 0.03). The PINK1/Parkin mitophagy pathway, Parkin (LME, slope = −0.96, *p* = 0.01) and pUb^Ser65^ (slope = −0.66, *p* = 0.05) were also significantly deficient in PD neurons compared to healthy controls.

The signalling proteins SIRT3 and TFAM were also significantly decreased in PD compared to healthy controls (*p* ≤ 0.05, Fig. 3b–d). The mitochondrial transcription factor, TFAM has been previously shown to be deficient in PD neurons using immunofluorescence^8^. SIRT3 the NAD^+^ dependent histone deacetylase, is one of the upstream regulator of TFAM and its deacetylase activity of SIRT3 impacts over 165 mitochondrial proteins^27^. The defect in the SIRT3/TFAM axis found in the PD neurons further supports interruptions in mitochondrial proteostasis within the neuronal MQC regulatory system.

To determine if these observations were unique to PD, we compared the effects observed in PD to neurons harbouring POLG mutations. POLG neurons showed significantly lower levels of a fission protein, DRP1 (slope = 1.20, *p* = 0.04) and the PINK1-phosphorylated mitofusin protein, MFN2 (slope = 0.96, *p* = 0.03, Fig. 3b–k). This difference was not observed between neurons with PD or mtDNA point mutations, indicating the impaired mitochondrial dynamics which can lead to the low capacity of buffering dysfunctional mitochondria is specifically associated with *POLG* mutations.

Our observations in single neurons (Fig. 3c–k) were recapitulated when calculating the average level of abundance per individual (Supplementary Fig. 2). This indicates that, despite the heterogeneity of individuals, the majority of signalling proteins showed a trend towards decreased abundance of tested MQC proteins in PD cases with the exception of DJ-1 and DRP1. Meanwhile, our analysis did not reveal significant differences in mitochondrial mass (measured using anti-Porin1/VADC1) by between PD and mitochondrial disease cases (mtDNA point mutations or POLG) or controls (Fig. 3l), nor in the abundance of test MQC proteins between neurons carrying a mtDNA point mutation and PD (Fig. 3b–k). In addition, multiple linear regression with interaction modelling^28,29^ revealed no significant impact of age (*p* = 0.78), post-mortem delay (*p* = 0.89) or sex (*p* = 0.86) on the protein level. This suggests the identified deficiencies in the MQC proteins were overwhelmingly attributed to disease-specific changes, consistent to our previous analysis in a small group of mitochondrial proteins in PD brains^23^.

### Correlation of MQC proteins in PD

**Fig. 4:**
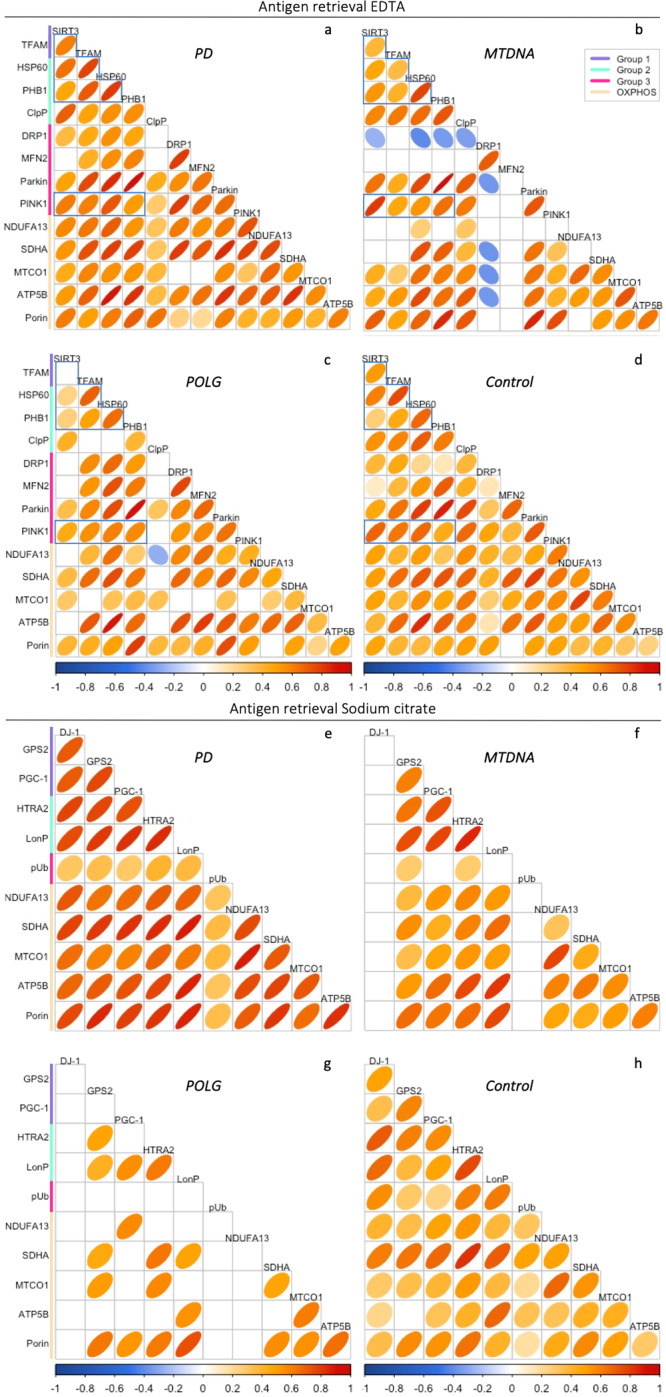
Single-neuron correlation analysis of mitochondrial MQC and OxPhos proteins. **a**–**h** Correlation matrices showing the strength of the association between tested proteins, basing on Spearman correlation analysis using single neuronal data. The colour and shape of the ellipse represent the Spearman’s rank correlation coefficient r of each paired protein (thinner ellipses represent stronger correlations); paired proteins showing no significant correlation were left blank (adjusted *p* > 0.0003, **a**–**d**; adjusted *p* > 0.0006, **e**–**h**).

The multiplexed nature of IMC enables the simultaneous interrogation of multiple proteins involved in the same pathway or process within the same sample. In this study, we utilised Spearman correlation analysis to investigate the relationships between protein abundances at the single-neuron level in PD, mitochondrial disease, and healthy control cases (Fig. 4a–h).

In healthy controls, proteins involved in the same pathway/process were significantly correlated, for example, Parkin and PINK1 (*r* = 0.68, *p* < 0.001, Fig. 4d) and mitochondrial proteases HTRA2 and LonP1 (*r* = 0.73, *p* < 0.001, Fig. 4d). These correlations were well maintained in both the PD and mitochondrial disease cases (*r* = 0.64–0.73, Fig. 4a–c). Corresponding with the intricate nature of mitochondrial adaptability, proteins from interacting pathways were also significantly correlated. For example, a strong positive correlation was found between Parkin and PHB1 in all groups (*r* = 0.83–0.95, Fig. 4a–d), which is in agreement with the recent discovery that the PHB complex is involved in Parkin-mediated mitophagy in cultured cells^30^. Similarly, the abundance of the ATP-dependent protease, LonP1 was highly correlated with ATP5B (POLG, *r* = 0.57; other tested groups, r = 0.75-0.87; Fig. 4e–h).

Overall, there are stronger correlations between the tested signalling proteins in PD neurons, compared to controls (Fig. 3). This suggests that the reduction of MQC proteins abundance might be synergistic in PD neurons. In particular, those signalling proteins that were identified as significantly decreased in PD, including PHB1, SIRT1, TFAM and HSP60 showed enhanced correlations compared to both healthy controls and mitochondrial cases. Similarly, the correlation of PINK1 with these four proteins was also increased in PD (frames, Fig. 4a–d).

### IMC detection of mtDNA-encoded OxPhos subunits

Subunits of all five OxPhos complexes were detected simultaneously with the signalling proteins Supplementary Figure 2a. In addition to our previously reported OxPhos panel^23^, we included an additional three antibodies which target specific mtDNA subunits to investigate changes in nuclear and mtDNA-encoded OxPhos subunits within each group (Supplementary Fig. 3a).

Both complex I subunits, NDUFA13 and MTND4 of complex I were highly correlated in all four groups (*r* = 0.73–0.85, *p* < 0.05), possibly due to the close association of assembly and function of this large respiratory complex. Complex V subunit, ATP5B and MTATP8 showed strong positive correlation in PD, POLG and control cases (*r* = 0.63–0.81, *p* < 0.05) but not in the mtDNA point mutation group (*r* = 0.09, *p* > 0.05). A weak correlation between complex III subunits, UqCRC2 and MTCyB was observed in PD (*r* = 0.11) and control (*r* = 0.24), such correlation was lost in both mitochondrial group (*p* > 0.05). These changes could reflect the mtDNA variants present in each group (Supplementary Fig. 3b).

### Determination of MQC elements responsive to OxPhos deficiency

**Fig. 5:**
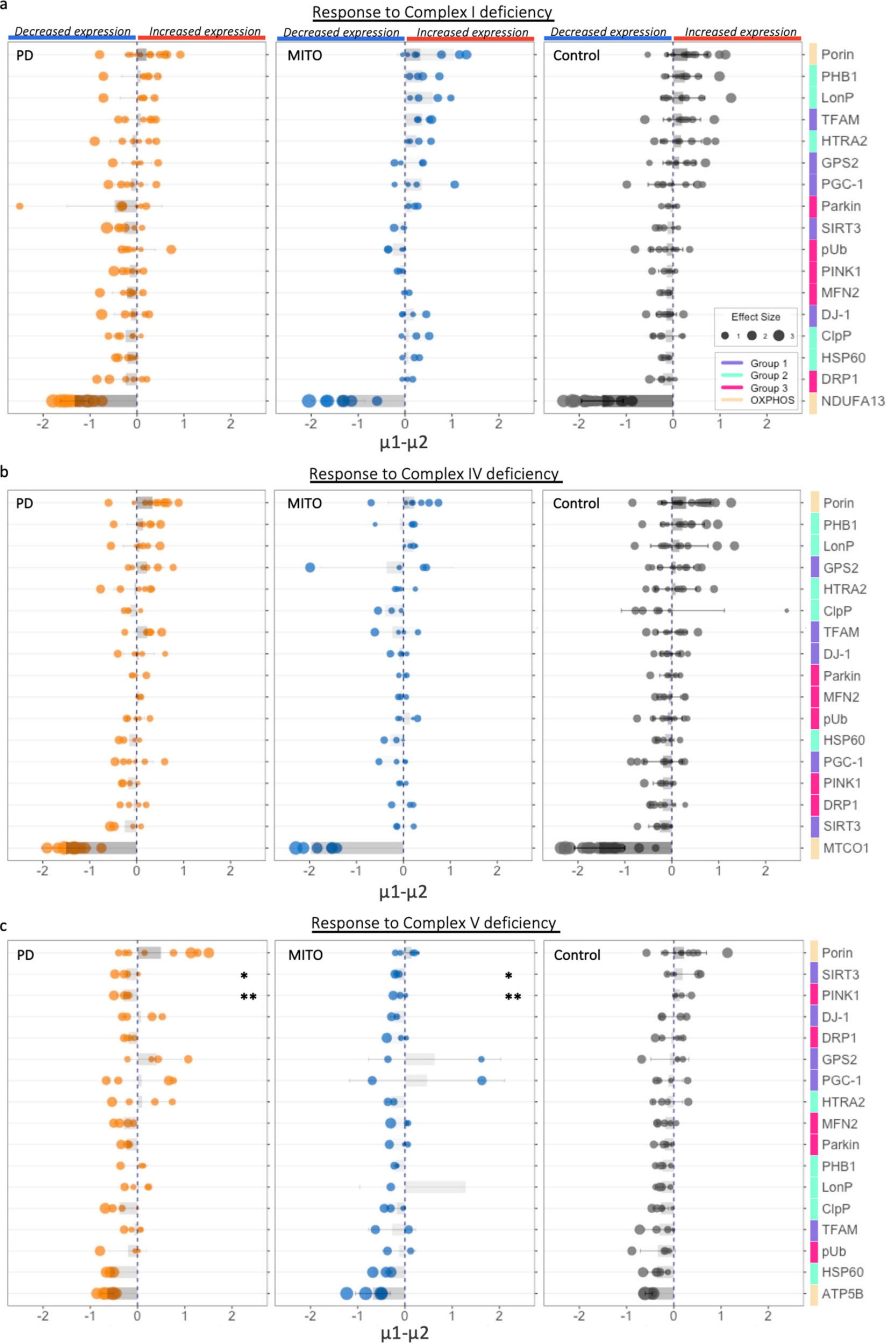
Investigation of the changes in signalling protein expressions in association with OxPhos deficiency. **a–c** SN neurons showing deficiency (that fell below the lower 10% limit of the prediction interval of the control) in complex I (NDUFA13), IV (MTCO1) or V (ATP5B) and those with normal expression were categorised as two subgroups within each individual case. Within each individuals, differences in the expression level of tested proteins between the two subgroups were analysed using Bayesian Estimation modelling (µ1, PD; µ2, Control)^31^. The Difference of Means (µ1–µ2) generated from the modelling for each tested signalling protein were summarised and ranked in descending order based on the control, for the three types of OxPhos deficiencies respectively. Comparison of these differences in the responsive proteins between PD, mitochondrial disease and control group were also performed (ANOVA, *p*, *≤0.05, **≤0.01), no significant difference was identified in the complex I and IV responses. Each dot represents an individual case; bars and lines represent the mean and SD; dot size shows the effect size.

Next, we investigate whether the changes in signalling proteins are associated with OxPhos deficiency (Fig. 5). The MQC proteins were ranked according to the degree of their changes to deficiency in either complex I (NDUFA13), complex IV (MTCO1) or complex V (ATP5B). Consistent with our previous IMC report^23,28^, OxPhos deficiency was defined based on the 80% prediction interval of controls. The ranking was performed using the Difference of Means (µ1–µ2, Bayesian Estimation Modelling^31^), where µ1 and µ2 represent deficient and normal neurons respectively^23^ (Fig. 5a, b). This modelling eliminates the effect of the varying number of neurons that fell into each subgroup within each individual, as it can account for such uncertainty in the estimated effect sizes, leading to more robust conclusions compared to traditional t-test^31^. In addition to the Difference of Means and effect sizes, other parameters from the comparison modelling including 95% highest density interval (HDI) and Region of Practical Equivalence (ROPE) were listed in Supplementary Table 2.

An Increased abundance of the tested MQC proteins (indicated by µ1–µ2 > 0) in OxPhos deficient neurons might indicate a positive change to maintain mitochondrial homoeostasis. Examining the differential responses in PD, mitochondrial disease and healthy control neurons would help to extend current knowledge of PD-specific disease pathogenesis and provide novel insights and targets for therapeutic and biomarker development. Our analysis identified two mitochondrial proteases, PHB1 and LonP1, which showed the strongest increase in response to complex I and IV deficiency in the healthy control (Fig. 5a, b). Similar increases were also observed in some mitochondrial disease (complex I, 4/4 cases; complex IV, 3/4) and PD cases (complex I/IV, 4/6 cases). However, due to the large individual diversity, such responses in mitochondrial proteases did not reach statistical significance between PD and control groups (ANOVA, *p* > 0.05).

Interestingly, changes in response to complex V deficiency showed a different pattern to complex I and IV **(**Fig. 5c). The two signalling factors, SIRT3 and PINK1 which did not show strong responses to complex I or IV deficiency, were significantly increased in complex V deficient neurons in control cases (SIRT3: µ1–µ2 = 0.52, effect size = 0.85; PINK1: 0.36,0.53; Fig. 5c). However, the reverse was seen in the PD and mitochondrial disease cases (effect size<0.25), showing a significant loss of response compared to the controls (ANOVA, *p* = 0.035, PD, *p* = 0.041, MITO, SIRT3; PD, *p* = 0.006, MITO, *p* = 0.008, PINK1). This indicates that the SIRT3-PINK1 pathway, as part of signal transduction cascade that regulate mitochondrial fusion, fission and mitophagy^32^ might fail to adapt to neuronal energy deficits

### Phosphorylated ubiquitin aggregation and increased mitochondrial proteases with α-Synuclein pathology

**Fig. 6:**
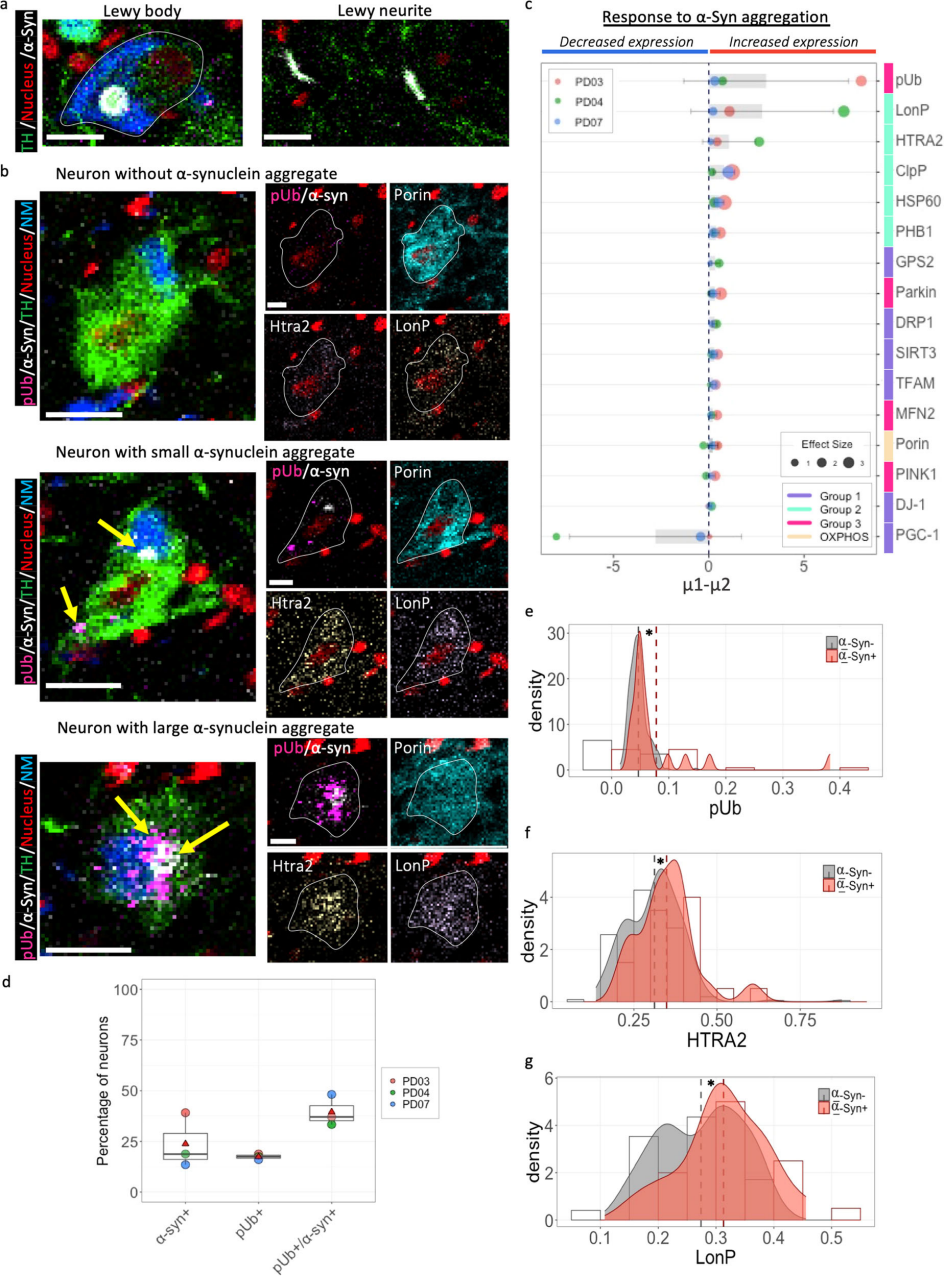
Changes in MQC proteins in PD neurons with α-Synuclein aggregation. **a** Example IMC images of anti-phosphorylated α-Synuclein ^Ser129^(α-Syn) antibody labelling in a TH-positive neuron, showing Lewy body within the neuronal soma and Lewy neurites. Scale bar, 20 µm. **b** To investigate the response of tested signalling proteins to α-Syn pathology, intra-individual analysis of changes in the protein abundance was performed between PD neurons with and without α-Syn aggregates (signal pixel intensity>5, the number of adjacent positive pixel > 3), using Bayesian Estimation modelling (µ1, α-Syn positive; µ2, α-Syn negative). Each dot represents an individual case (Number of neurons analysed, PD03, 04 &07, *n* = 44, 39 &11); bars and lines represent the mean and SD; dot size shows the effect size. **c** Corresponding to the response analysis, three TH-positive neurons selected from PD03 (male, 80 yrs) with varying degree of α-Syn aggregation were demonstrated, alongside signals from the three signalling proteins, phosphorylated ubiquitin^Ser65^ (pUb), LonP and HTRA2 which showed the highest level of increases in α-Syn positive neurons, compared to the α-Syn negative ones among tested proteins. Scale bar, 20 µm. **d** Proportion of neurons with α-Synuclein or/and pUb aggregates were calculated to support the observation of their co-existence within the PD neurons. Each dot represents an individual case; boxplots show median and mean (triangle) 25th and 75th percentile, with whiskers indicating 95th upper/lower interquartile range. **e**–**g** LMM analysis further confirmed the changes of pUb, LonP and HTRA2 to α-Syn pathology at a group level (6/8 PD cases, *n* = 121 neurons analysed; *p* < 0.05).

In this study, the relationship between changes in MQC proteins and α-Synuclein (α-Syn) pathology was investigated using Bayesian modelling (Fig. 6a). α-Syn aggregates were identified using an antibody against phosphorylated α-Syn^Ser129^, a form of α-Syn typically associated with disease status and elevated in PD brain tissue^33,34^. Protein aggregation was statistically defined based on a signal pixel intensity >5, with the number of adjacent positive pixel >3, this analysis is therefore included neurons containing both Lewy bodies and more diffuse accumulations of α-Syn.

Through ranking of the Difference of Means (µ1–µ2, µ1- α-Syn positive; µ2- α-Syn negative neurons), we identified the top three signalling factors that showed significant increases to α-Syn pathology: pUb^Ser65^ (µ1–µ2, 0.31-8; effect size, 0.48–0.90), LonP1 (0.23–7.09; 0.41–1.07) and HTRA2 (0.11–2.64; 0.20–0.68) in neurons with α-Syn aggregates, compared to those ones without (Fig. 6b, c and Supplementary Table 3). Intracellular aggregates of pUb^Ser65^ were observed in some PD neurons (19.8 ± 0.65% Fig. 6d) and their co-existence with phosphorylated α-Syn aggregates (37.8 ± 5.3%) were also noted. This finding is consistent which a recent report from a Lewy body disease cohort^35^. Interestingly, the intraneuronal co-existence of pUb^Ser65^ and α-Syn was also detected in a POLG case (POLG02, Supplementary Fig. 4), supporting the association of protein aggregation with mitochondrial defects. However, a larger cohort study is required to benefit statistical comparison.

However, due to the limited number of SN neurons which contained intracellular α-Syn aggregates, 3/8 PD cases (*n* = 94 neurons analysed) were eligible for Bayesian Estimation modelling (*n* ≥ 3 per subgroup is the minimum requirement). The findings of pUb^Ser65^, LonP1, HTRA2 and PGC-1α were further confirmed using linear mixed effects modelling for the significant changes (*p < 0.05, 6/8 PD cases analysed, Fig. 6e–g).

On the other hand, significant decrease in PGC-1α level in neurons with α-Syn aggregates (−8 to −0.43; 0.27–0.58), which could be due to nuclear α-Syn binding to PGC-1α promotor and downregulate its expression^36,37^. Together, these results support that synuclein pathology affect both intramitochondrial proteases and organelle degradation. However, inclusion of a larger sample size and high-resolution microscopy is warranted for further validation.

## Discussion

In this study, we used a panel of antibodies was designed to investigate targets associated with the maintenance of a healthy mitochondrial population, including mitochondrial dynamics, biogenesis and the UPR^mt^. Many of targets have been previously linked with the vulnerability of SN neurons and the pathogenesis of PD in either sporadic or familial, early-onset PD.

The degradation of mitochondria through mitophagy has been linked with PD through numerous studies^2^, though this process has not been examined in detail in neuronal tissue from people with Parkinson’s. We observed significant decreases in both PINK1 and Parkin with the retention of a close correlation between the two protein PD neurons, compared to controls. However, it is worth noting that the PINK1/Parkin ubiquitination-directed mitophagy relies largely on PINK1’s phosphorylation activity instead of protein abundance^38,39^. PINK1 phosphorylates ubiquitin and Parkin’s ubiquitin-like domain at pUb^Ser65^^40^, both are required for the activation of Parkin’s E3 ubiquitin ligase^41^ to activate mitophagy. Our IMC data provides evidence that in post-mortem PD SN dopaminergic neurons, the PINK1-Parkin mediated amplifying mechanism which renders mitophagy more efficient is compromised, based on the synergetic decrease in levels of PINK1, Parkin and pUb^Ser65^. Previous studies have suggested that the expression of PINK1 mRNA is maintained in PD^42^, which suggests that it may be the post-translational modifications of this protein^43^ or the UPS-mediated degradation^44^ that is affected in these PD neurons. However, due to the nature of this multiplex immunostaining assay in which all targeted protein antigens are retrieved in one way on FFPE sections, we are limited to being able to examine protein level without simultaneous investigation of different active/reactive forms.

In addition to the decreased protein level in the PINK1-Parkin mitophagy pathway, we observed intracellular aggregation of pUb^Ser65^ within the DA neurons of PD. This aggregation can be triggered by mitochondrial damage^45^ (Supplementary Fig. 1) and mainly presented in the α-Syn positive neurons of PD, suggested the perturbations in the PINK1/Parkin pathway^46^, possibly associated with synuclein pathology^35^. The aggregation of pUb^Ser65^ may be a direct result of increased mitochondrial damage or disrupted UPS function that affects its turnover, or could be in combination with blockage of lysosomal clearance machinery of mitochondrial-targeted autophagosomes^47^. This is consistent with reports of increased dysfunctional (nitrosylated or insoluble) Parkin^48,49^ and impaired Parkin E3 ligase ubiquitin ligase activity by α-Syn^50^ in PD brains. These findings would require further investigation of the interactive relationship between varying forms of PINK1, Parkin and pUb^Ser65^ to help unravelling the role of mitophagy in the formation of pUb^Ser65^ and α-Syn aggregation. Autophagy receptors and component proteins of the autophagosome, such as Optineurin (Optn) and LC3 would also need to be examined to determine the impact of these changes on long-lived protein turnover^51^.

Polyubiquitin linkages of pUb^Lys48^ and pUb^Lys63^ attached to and ubiquitinate α-Syn to facilitate its clearance, but this may also promote the formation of aggregates if the proteasome is overwhelmed or impaired^52,53^. PUb^Ser65^ can affect the structure and assembly of ubiquitin chains^54^ and its aggregation has been reported in ageing and PD brains^45^, suggesting its potential as a disease biomarker. Our findings of the co-existence of pUb^Ser65^ and α-Syn aggregates, alongside an increase of mitochondrial proteases (LONP1 and HTRA2) in PD neurons provided post-mortem evidence supporting the link between synuclein pathology and mitochondrial defects. However, the cause and consequence of these two key events in the pathogenesis of PD need to be further elucidated.

We also examined the abundance of proteins which are integral to the correct processing of essential mitochondrial proteins, detecting significant decreases in the level of two tested mitochondrial chaperones in PD, HSP60 and PHB1. HSP60 mediates correct intramitochondrial protein folding activity, as a co-chaperone with HSP70 for protein translocation and stress protection^55^. While PHB1, a key component of the PHB complex maintains protein stabilisation via inhibition of mitochondrial proteases and aids the formation of mitochondrial respiratory chain (RC) supercomplexes^56^. An upregulation of HSP60 has been previously characterised in an MPTP treated cell model^57^ and in neurons as a response to hippocampal stress in an epileptic rodent model^58^. However, our study is the first to describe its deficiency in SN DA neurons in PD, supported by a recent description of links between HSP chaperones and synuclein pathology^59^. It has been found that α-Syn interferes with the intramitochondrial function of the HSP10/HSP60 complex through sequestration of HSP10 in the cytosol. Meanwhile, the characterisation of decreased PHB1 abundance in the DA neurons of PD is consistent with previous findings of low PHB levels in MPTP mouse and cell models^60^, and in the frontal cortex of PD patients^61^. Declines in HSP60 and PHB1 would both cause an increase in superoxide dismutase 2 (SOD2) generation^59,60^, suggesting that neuroprotection against oxidative stress is compromised in these PD neurons.

Dopaminergic neurons in the SN are particularly vulnerable to neurodegeneration in PD. Their vulnerability involves proteostatic dysfunction and changes in intracellular calcium dynamics, alongside increased susceptibility to mitochondrial oxidative stress^62^. Despite this, previous IF/IHC studies have identified no positive correlation between the severity of mitochondrial RC deficiency and α-Syn expression^63,64^. Our IMC analysis links mitochondrial OxPhos dysfunction and synuclein pathology with changes in the levels of proteins essential for mitochondrial proteostasis. Intra-individual comparison demonstrated increased abundance of PHB1, LonP1 and HTRA2 in neurons carrying complex I/IV deficiency from neurologically healthy aged individuals, indicating a proteosomal response to RC deficiency in these SN neurons (Fig. 5). Though such increases were also seen in the majority of tested PD cases, a declining trend was found in the average level of increase in the PD group, compared to healthy controls and mitochondrial disease cases. This may suggest that loss of mitochondrial proteasomal adaptability in response to increased oxidative damage is more associated with the PD process rather than it is a consequence of a primary mitochondrial defect.

An increase of LonP1 and HTRA2 abundance was found in the SN DA neurons with such aggregates compared to those without in PD (Fig. 6). Regardless of the number of cases sampled (a total of *n* = 94 neurons were analysed), these data provide some evidence to support an enhanced intramitochondrial protease activity under the stress of synuclein pathology. Though the underlying mechanism requires further elucidation, our post-mortem results confirm that synuclein regulation and an impaired MQC regulatory system could be mutually affected and the enhanced mitochondrial proteosomal expression could perhaps be a protective mechanism against α-Syn aggregation. Future studies would rely on high-resolution microscopic investigation of the relationship between high mitochondrial protease abundance and the various forms of α-Syn aggregate, including both Lewy bodies and a more diffuse accumulation of α-Syn. A recent ultrastructural study reported the presence of dystrophic and fragmented mitochondria within Lewy bodies, including around their corona^65^. Characterising different effects of morphologically distinct α-Syn aggregates on intramitochondrial proteases could provide further insights on the interaction of synuclein pathology and mitochondrial homoeostasis, and its consequential exacerbation of neuronal susceptibility to aged-related oxidative stress.

A further key finding is the low level of SIRT3, a mitochondrial deacetylase in SN neurons in PD. It is notable that SIRT3 was increased in response to complex V (ATP5B) deficiency in control SN neurons, but that this response was not observed in PD neurons. (Fig. 5c). SIRT3 regulates mitochondrial metabolism, supports responses to mitochondrial dysfunction and has been previously linked to neuroprotection^27^. Here, the SN neuronal response of increased SIRT3 levels to ATP synthase deficiency, detected in the healthy control cases did not present in those with complex I/IV deficiency. SIRT3 directly deacetylates complex I and IV subunits with a lack of evidence of its role in complex V deacetylation in neurons^66^. As a powerful metabolic sensor, the increase of SIRT3 in complex V deficient neurons may be driven by bioenergetic alterations. SIRT3 activity is coordinated with increased mitochondrial NAD^+^ production, and on the other hand, is inhibited by an increase in the NADH/NAD^+^ ratio as a negative feedback mechanism. It has been proposed that the protective effect of SIRT3 was triggered by moderate increase of ROS production, whilst a high level of oxidative stress could eliminate this neuroprotective response^27^. Neurons with complex I/IV defects may be exposed to a higher level of oxidative stress in comparison to those with complex V deficiency, hence the responsive changes in SIRT3 expression might not occur. Emerging evidence from human cell culture and animal models has highlighted the roles of SIRT3 in neuroprotection due to the regulatory capacity in the maintenance of mitochondrial homoeostasis, anti-oxidation and biogenetics in response to increased cellular stress, particularly during ageing^67–69^. A loss of SIRT3 protein abundance and response to dysfunctional ATP synthesis in these PD neurons undoubtedly affects their ROS defence and adaption to energy deficits that are associated with both ageing^70^ and α-Synuclein pathology^71^, leading to increased neuronal susceptibility to the disease. This is supported by the observation in SIRT3 knockout mice of an exacerbation of MPTP-induced DA deficits in the nigrostriatal pathway^72^.

In this study we used IMC to interrogate the levels of proteins in three major groups associated with the regulatory machinery of MQC. We are mindful of the complexity of these regulatory systems, including the intricate interactions between pathways and multi-functional roles of each signalling protein. Mitophagy undoubtedly plays a central role in the regulation of MQC, however, activation of PINK1/Parkin triggers multiple mechanisms to synergistically regulate mitochondrial homoeostasis, including increased fission, local synthesis of OxPhos proteins and new organelles, and import of mitochondrial proteins^41^. We reveal that SN neurons in PD cases show a generalised reduction in MQC proteins. This study suggested disrupted mitochondrial turnover and the maintenance of mitochondrial proteostasis in PD neurons. This could exacerbate the impact of oxidative phosphorylation defects and aged-related oxidative stress, leading to early neuronal death in PD (Fig. 1a).

## Methods

### Ethical approval and consent to participate

All post-mortem midbrain sections used in this study were obtained from the Newcastle Brain Tissue Resource (NBTR, https://nbtr.ncl.ac.uk/) with written consent given by donors or their next of kin for research. Ethical approval for the use of all tissues was provided by the National Health Service Local Research Ethics Committee and adhered to the Medical Research Council’s (MRC) guidelines on the use of human tissue in medical research.

### Tissue cohort

Eight clinically and pathologically diagnosed (Braak stage of Lewy pathology >III) idiopathic PD cases were included in this study. They were compared to six mitochondrial disease cases as disease controls, to help identify changes specific to PD pathology: alongside 11 individuals without neurological disease as healthy controls (Fig. 2a). Three mitochondrial disease cases carried maternally inherited mtDNA mutations affecting mitochondrial respiratory chain such as *m.3243A*>*G* and *m.8322A*>*G* (herein referred to as MTDNA, *n* = 3), with the remainder carrying *POLG* mutations associated with multiple mtDNA deletions (herein referred to as POLG, *n* = 3). To allow comparison and age-matching with both disease groups, we also included two younger healthy controls (18 and 19 years old). Details for individual cases are listed in Supplementary Table 1.

### Imaging mass cytometry

Formalin-fixed paraffin-embedded (FFPE) sections (5 µm) of upper midbrain at the level of the superior colliculus were subjected to two customised IMC antibody panels based on the optimal antigen retrieval method for each antibody (Table 1). Nine antibodies were assessed in the presence of EDTA (pH = 8) and six were assessed in the presence of sodium citrate buffer (pH = 6). Signalling antibodies were selected based upon validation by either the supplier based on knockout or knockdown models, or by the authors using siRNA knockdown models, western blotting, immunofluorescence, and immunohistochemistry, as summarised in Table 1. The anti-pUb, anti-Parkin, anti-HSP60 and anti-DJ-1 antibodies were further optimised using an acute mitochondrial-damaged cell model, which showed mitochondrial-dependent changes in the signals (Supplementary Fig. 1). Both experiments also included antibodies targeting OxPhos subunits, a marker for mitochondrial mass (Porin/VDAC1), a DA neuron marker (TH) and a nuclear marker (Histone H3) as previously reported^23^ (Table 1). Immunolabelling of the antibody panels was performed based on a protocol adapted from^23,73^.

**Table 1:** Information of antibodies used in the study.

Primary antibody (Clone/RRID)	Description	Supplier	Lot	Metal tag	Final dilution	Antigen retrieval	Antibody validation
Signalling proteins markers							
Anti-DJ-1 (D29E5; AB_11179085)	Parkinsonism-associated deglycase-7; PARK7	5933BF Cell Signalling	^5933*bf*^	^149^Sm	1:50	Sodium citrate, pH = 6	KO validated by the manufacture; PMID: 32051471
Anti-GPS2	G protein pathway suppressor 2	*	^/^	^155^Gd	1:50	Sodium citrate, pH = 6	*
Anti-PGC-1α (Polyclonal; AB_2268462)	Peroxisome proliferator-activated receptor gamma coactivator 1-alpha	AB3243 Millipore	^3111137^	^171^Yb	1:50	Sodium citrate, pH = 6	KO validated by PMID: 28611589 & 31634150
Anti-SIRT3 (Polyclonal; AB_10861832)	NAD-dependent protein deacetylase sirtuin-3, mitochondrial	ab86671 Abcam	^GR291825-4^	^159^Tb	1:50	EDTA, pH = 8	KO validated by the manufacture;
Anti-TFAM (18G102B2E11; AB_10900340)	Transcription factor A, mitochondrial	ab119684 Abcam	^GR3292863-3^	^156^Gd	1:50	EDTA, pH = 8	Validated by the MitoSciences^®^
Anti-HSP60 (24/HSP60 (RUO)/ AB_399008)	60 kDa heat shock protein, mitochondrial	611562 BD Transduction	^611563^	^152^Sm	1:50	EDTA, pH = 8	Validated using culture model (Supplementary Fig. 1)
Anti-PHB1 (Polyclonal; AB_823689)	Prohibitin1	2426BF Cell Signalling	^2426BF^	^171^Yb	1:100	EDTA, pH = 8	KD validated by the manufacture
Anti-ClpP (Polyclonal; AB_1078538)	ATP-dependent ClpP protease proteolytic subunit, mitochondrial	HPA010649 Sigma	^C115652^	^162^Dy	1:50	EDTA, pH = 8	KO validated by the manufacture; PMID: 23851121
Anti-HTRA2 (Polyclonal; AB_2280094)	Serine protease HTRA2, mitochondrial	AF1458 R&D systems	^IJO0917101^	^165^Ho	1:50	Sodium citrate, pH = 6	KO validated by PMID: 25531304 & 19443712
Anti-LonP1 (Polyclonal; AB_1079695)	Lon protease homologue, mitochondrial	HPA002192 Sigma	^/^	^172^Yb	1:50	Sodium citrate, pH = 6	KO validated by the manufacture
Anti-DRP1 (D8H5; AB_11178938)	Density-regulated protein	5391BF Cell Signalling	^5391BF^	^168^Er	1:50	EDTA, pH = 8	KD validated by the manufacture; PMID: 33520735
Anti-MFN2 (6A8;AB_2142629)	Mitofusin-2	ab56889 Abcam	^GR3224958-1^	^165^Ho	1:50	EDTA, pH = 8	KO validated by PMID: 27228353
Anti-Parkin (Polyclonal; RRID:AB_2892811)	E3 ubiquitin-protein ligase; PARK2	NBP2-41287 Novus	^7903-1304^	^172^Yb	1:50	EDTA, pH = 8	Validated using culture model (Supplementary Fig. 1)
Anti-PINK1 (Polyclonal; AB_10127658)	Serine/threonine-protein kinase, mitochondrial	BC100-494 Novus	^M^	^167^Er	1:50	EDTA, pH = 8	KO validated by PMID: 23256036; 30258205
Anti-Phospho Ub (Polyclonal; AB_2858191)	Ubiqutin, Ser65 phosphorylated; pUb^Ser65^	ABS1513 Millipore	^3117322^	^167^Er	1:200	Sodium citrate, pH = 6	Validated using culture model (Supplementary Fig. 1)
OXPHOS markers							
Anti-MTND4 (9E4-2D8)	Complex I subunit (mtDNA encoded)	*	^/^	^164^Dy	1:50	EDTA, pH = 8	*
Anti-NDUFA13 (6E1BH7; AB_10863178)	Complex I	ab110240 Abcam	^GR3229012-1^	^169^Tm	1:25	Both	Validated by the MitoSciences^®^
Anti-SDHA (2E3GC12FB2AE2 ;AB_301433)	Complex II	ab14715 Abcam	^GR3229010-1^	^153^Eu	1:50	Both	Validated by the MitoSciences^®^
Anti-UqCRC2 (13G12AF12BB11; AB_2213640)	Complex III	ab14745 Abcam	^GR3229018-1^	^174^Yb	1:50	EDTA, pH = 8	Validated by the MitoSciences^®^
Anti-MTCyB (5B3-6E3)	Complex III subunit (mtDNA encoded)	*	^/^	^115^In	1:50	EDTA, pH = 8	*
Anti-MTCO1 (1D6E1A8; AB_2084810)	Complex IV subunit (mtDNA encoded)	ab14705 Abcam	^GR229009-1^	^158^Gd	1:50	Both	Validated by the MitoSciences^®^
Anti-ATB5B (3D5; AB_301438)	Complex V	ab14730 Abcam	^GR3236125-2^	^170^Yb	1:50	Both	Validated by the MitoSciences^®^
Anti-MTATP8 (1G3-1H11-1C3)	Complex V subunit (mtDNA encoded)	*	^/^	^161^Dy	1:50	EDTA, pH =8	*
Cell markers							
Anti-VDAC1/Porin1 (20B12AF2; AB_443084)	Voltage-dependent anion-selective channel protein 1; Mitochondrial mass marker	ab14734 Abcam	^GR3229011-1^	^147^Sm	1:100	Both	Validated by the MitoSciences^1^
Anti-TH (TH-16; AB_477569)	Tyrosin hydroxylase; Dopaminergic marker	SAB4200697 Sigma	^SLBT6779^	^176^Yb	1:200	Both	/
Anti-Histone H3 (24HC2LC12; AB_2532490)	Histone H3; Nuclear marker	701517 Invitrogen	^2010233^	^142^Nd	1:400	Both	/
Anti-alpha synuclein (EP1536Y; AB_869973)	phospho S129; alpha-synuclein marker	ab209422, Abcam	^GR3243223-1^	^145^Nd	1:400	Sodium citrate, pH = 6	/

*Antibodies were provided by collaborators: Anti-GPS2 -Dr. Valentina Perissi (Boston University, USA); Anti-MTND4, MTCYB and MTATP8 -Dr. Michael F. Marusich. In prior to the IMC panel design and metal-conjugation, the signalling antibodies were tested using immunofluorescent assay in prior to the IMC panel design, using FFPE brain and/or frozen muscle sections to confirm their binding specificity and mitochondria-associated localisation (these images are available at 10.25405/data.ncl.22005623). The mitochondrial mass and OXPHOS markers have been well-validated and reported in previous studies using immunostaining/immunoblotting on several tissues, including brain^8,78^, muscle^79,80^ and other tissues^81–83^.

IMC detection and image reconstruction were performed using a Hyperion™ imaging system coupled to a Helios™ mass cytometer (Fluidigm). IMC images were visualised and exported as tiffs (MCD viewer, Fluidigm) and analysed using QuPath 0.2.3^74^. Individual DA neurons within the SN (including SNpc and SNpr) were segmented based on the presence of the neuronal (TH-positive), nuclear and neuromelanin markers; the mean pixel intensity per cytoplasmic area of each neuron was extracted for statistical analysis (Fig. 2).

### Statistical analysis

Statistical analysis was performed using R (v3.6.128^75^. Original pixel intensity values acquired from IMC images were natural log transformed and converted into z scores based on the entire control dataset for normalisation. Individual neurons were defined as OxPhos deficient if they fell below the lower limit of the 80% prediction interval of the control linear regression against mitochondrial mass (Fig. 5), as described previously^23^. The number of neurons available for analysis varied between each case (*n* = 60 ± 38 neurons per case), due to the varying levels of neuronal degeneration of each individual case. To account for individual variation, statistical differences between groups were quantified utilising both linear mixed effects modelling (LME)^26^ (Fig. 3b–l) and the ANOVA with Dunnett’s post-hoc tests (Fig. 5, Supplementary Fig. 2), using the PD group as a reference. Specifically, LME was employed for single-neuron analysis (Fig. 3b–l), whilst the ANOVA was used for analysis of the mean per individual (Fig. 5, Supplementary Fig. 2). As our previous IMC study reported^28^, LME is advantageous over ANOVA for single-neuron data analysis as it accounts for the variations between individuals, which can affect group-level comparisons. Spearman’s correlation, reported with adjusted p values, was performed to examine the relationship between tested proteins within each group (Fig. 4). We also used Bayesian Estimation (cases with n < 3 in either of the subgroups were not included), BEST package^31^ (Figs. 5 and 6), to described how each of the tested proteins respond to OxPhos deficiency/α-Syn, as previously reported^23,28^. Multiple linear regression with interaction modelling^76,77^ was used to determine the effects of age/post-mortem delay/sex and disease on protein level^28^.

### Reporting summary

Further information on research design is available in the Nature Research Reporting Summary linked to this article.

### Supplementary information

Supplementary Material

Reporting summary

## Data Availability

Raw datasets for this study available in the ‘Figshare’ repository (10.25405/data.ncl.22005623). Data are available under the terms of the Creative Commons Attribution 4.0 International license (CC-BY 4.0).
