# Supplementary material for: Parkinson’s disease neurons exhibit alterations in mitochondrial quality control proteins
Source: NPJ Parkinsons Dis. 2023 Aug 8;9:120. doi: 10.1038/s41531-023-00564-3 (PMC10409763; doi:10.1038/s41531-023-00564-3)
Supplement: Supplementary file 1 — Supplementary Material [file 41531_2023_564_MOESM1_ESM.pdf]

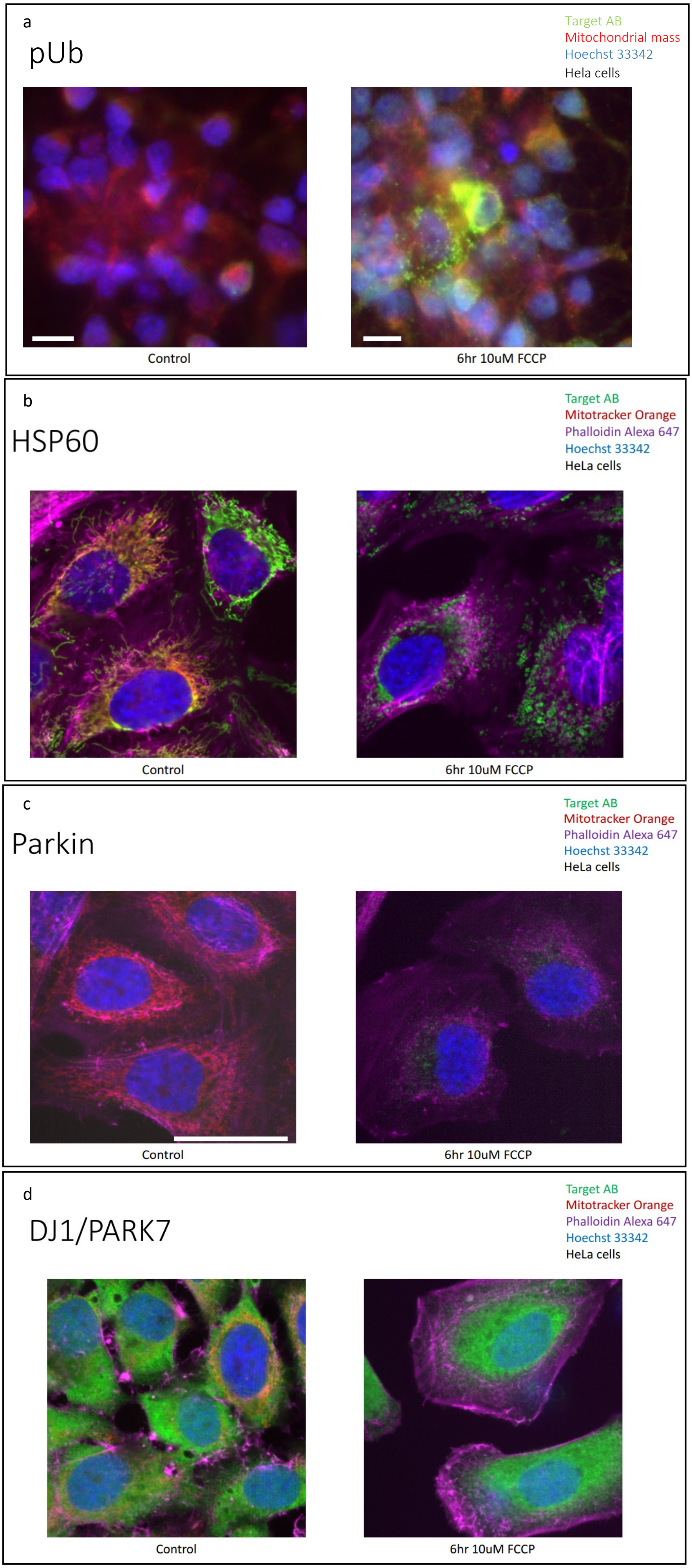
a
pUb
Target AB
Mitochondrial mass
Hoechst 33342
Hela cells
Control
6hr 10uM FCCP
b
HSP60
Target AB
Mitotracker Orange
Phalloidin Alexa 647
Hoechst 33342
HeLa cells
Control
6hr 10uM FCCP
c
Parkin
Target AB
Mitotracker Orange
Phalloidin Alexa 647
Hoechst 33342
HeLa cells
Control
6hr 10uM FCCP
d
DJ1/PARK7
Target AB
Mitotracker Orange
Phalloidin Alexa 647
Hoechst 33342
HeLa cells
Control
6hr 10uM FCCP

Supplementary fig. 1. Validation of the anti-pUb, anti-Parkin, anti-HSP60 and anti-DJ-1 antibodies using an acute mitochondrial-damaged cells model**.** Anti-pUb and anti-Parkin antibodies showed an increased signal (green), whilst the other two antibodies showed the opposite in mitochondrial-damaged HeLa cells (formalin-fixed) induced by carbonyl cyanide-p-trifluoromethoxy phenylhydrazone (FCCP, 10μm, 6hrs). This supports the binding specificity of these antibody, allowing the detection of changes associated to mitochondrial damage. Scale bar, 10μm.

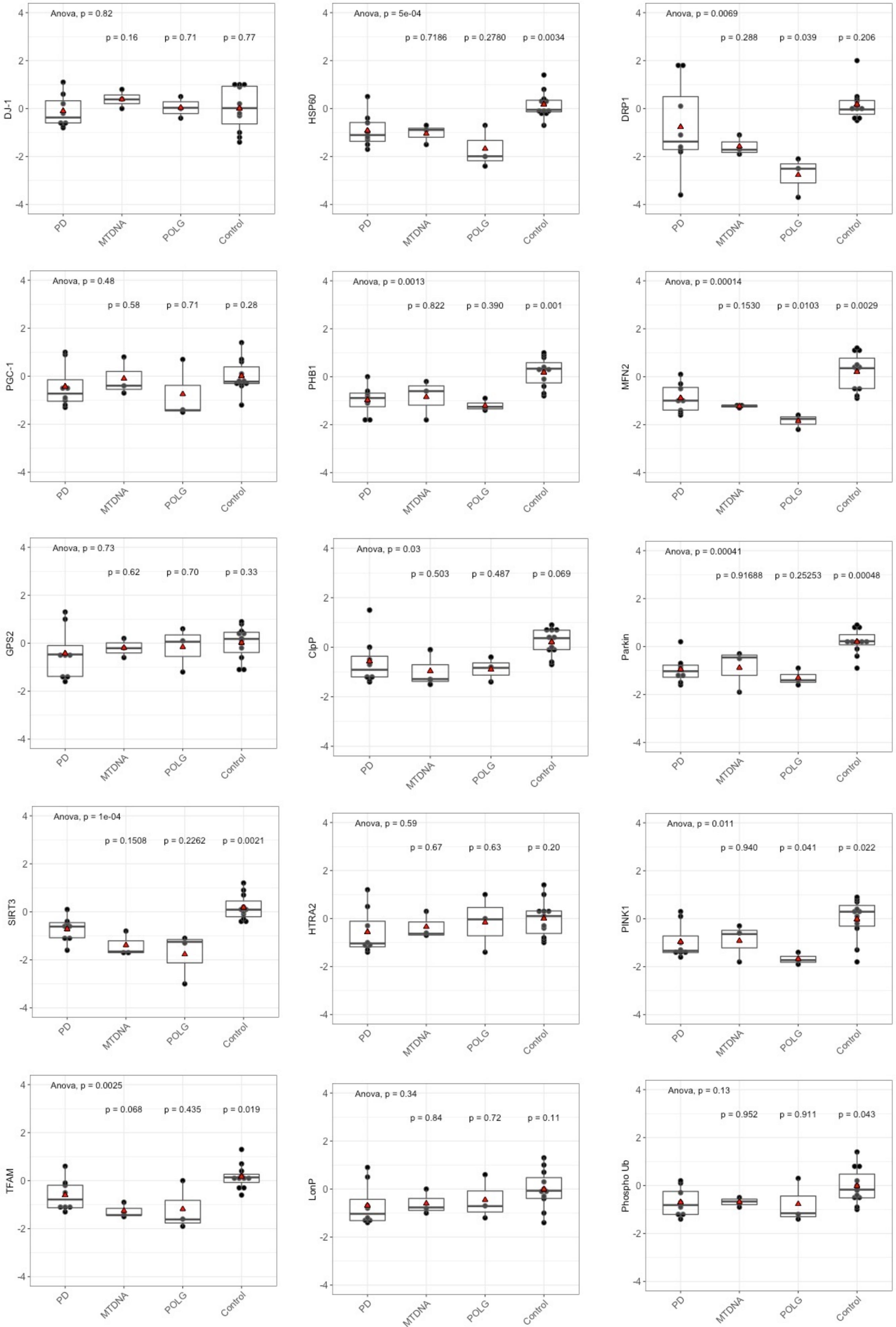
DJ-1
Anova, p = 0.82
p = 0.16
p = 0.71
p = 0.77
HSP60
Anova, p = 5e-04
p = 0.7186
p = 0.2780
p = 0.0034
DRP1
Anova, p = 0.0069
p = 0.288
p = 0.039
p = 0.206
PGC-1
Anova, p = 0.48
p = 0.58
p = 0.71
p = 0.28
PHB1
Anova, p = 0.0013
p = 0.822
p = 0.390
p = 0.001
MFN2
Anova, p = 0.00014
p = 0.1530
p = 0.0103
p = 0.0029
GPS2
Anova, p = 0.73
p = 0.62
p = 0.70
p = 0.33
ClpP
Anova, p = 0.03
p = 0.503
p = 0.487
p = 0.069
Parkin
Anova, p = 0.00041
p = 0.91688
p = 0.25253
p = 0.00048
SIRT3
Anova, p = 1e-04
p = 0.1508
p = 0.2262
p = 0.0021
HTRA2
Anova, p = 0.59
p = 0.67
p = 0.63
p = 0.20
PINK1
Anova, p = 0.011
p = 0.940
p = 0.041
p = 0.022
TFAM
Anova, p = 0.0025
p = 0.068
p = 0.435
p = 0.019
LonP
Anova, p = 0.34
p = 0.84
p = 0.72
p = 0.11
Phospho Ub
Anova, p = 0.13
p = 0.952
p = 0.911
p = 0.043
PD
MTDNA
POLG
Control

Supplementary fig. 2. Comparison of the mean level of each MQC protein expression between four groups (ANOVA test with Dunnett's post-hoc tests)**.** Each data point represents an individual case; triangles represent the mean and bars show the median and IQR.

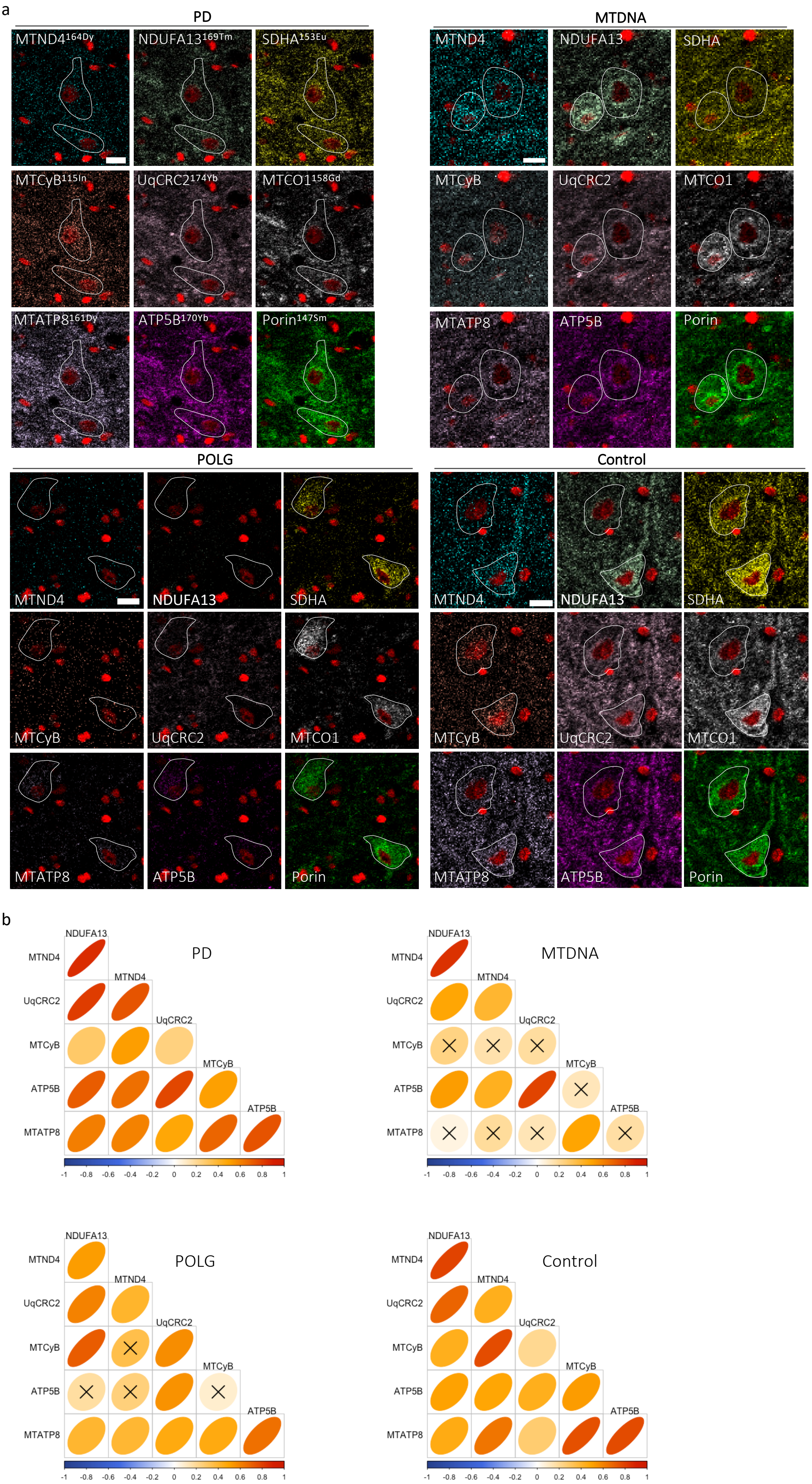

a
PD
MTND4 164Dy
NDUFA13 169Tm
SDHA 153Eu
MTCyB 115In
UqCRC2 174Yb
MTCO1 158Gd
MTATP8 161Dy
ATP5B 170Yb
Porin 147Sm
MTDNA
POLG
Control
MTND4
NDUFA13
SDHA
MTCyB
UqCRC2
MTCO1
MTATP8
ATP5B
Porin
b
PD
MTDNA
POLG
Control
NDUFA13
MTND4
UqCRC2
MTCyB
ATP5B
MTATP8
-1 -0.8 -0.6 -0.4 -0.2 0 0.2 0.4 0.6 0.8 1

Supplementary fig. 3. Single-neuron correlation analysis of nuclear and mtDNA encoded OxPhos subunits. a. IMC images of OxPhos subunits that were detected simultaneously within the neurons demonstrated in **Fig 2** and from a case with mtDNA point mutation (MTDNA01, male, 52yrs) in the EDTA batch. Scale bar, 20µm. **b.** Correlation matrices showing the closeness of association between each OxPhos subunit using single neuron data. Each block represents the correlation coefficient r values of each paired protein; X represents the pairs with no significant correlation (Spearman's correlation, adjusted $p=0.003$).

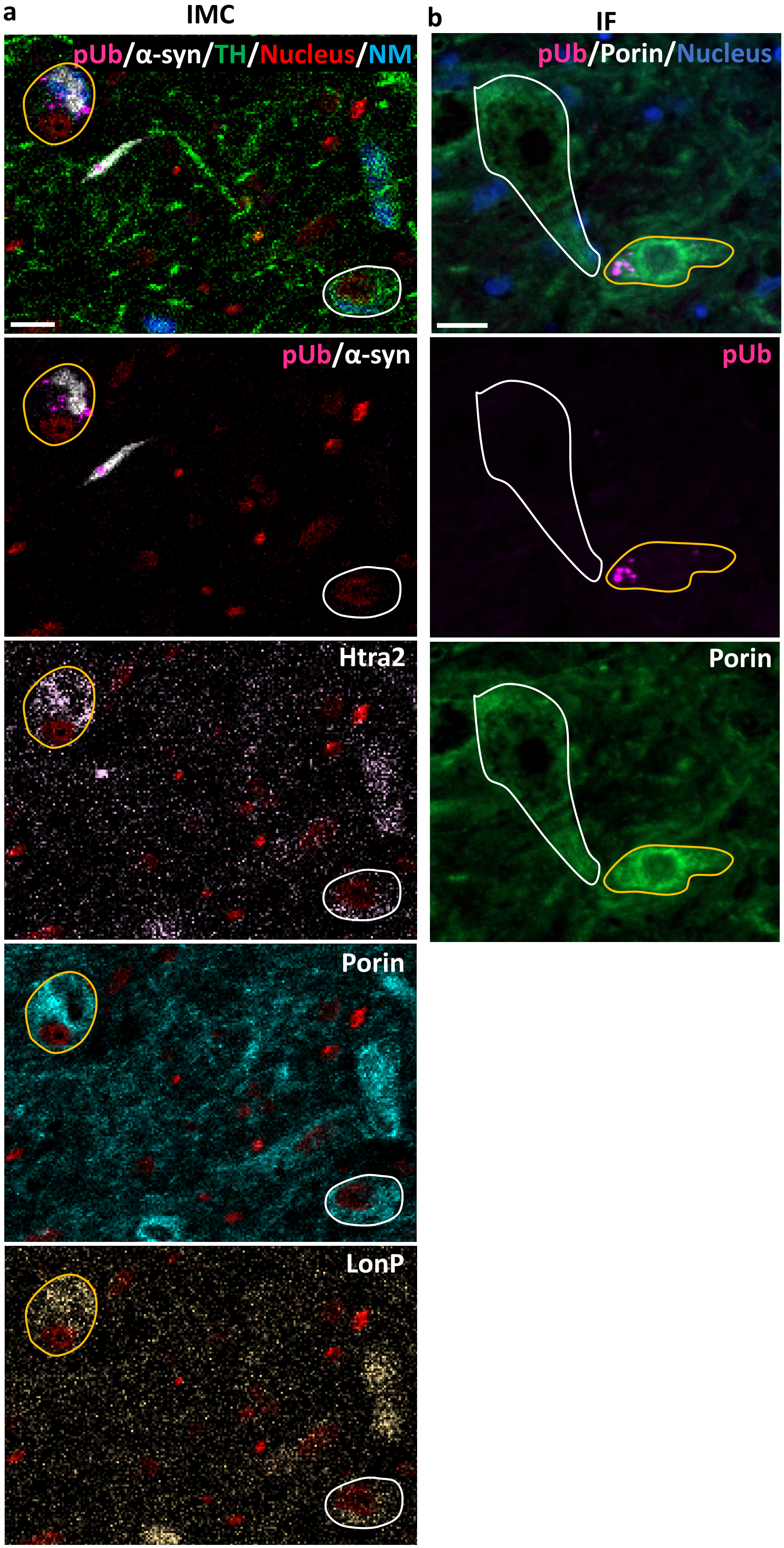

a
IMC
pUb/α-syn/TH/Nucleus/NM
pUb/α-syn
Htra2
Porin
LonP
b
IF
pUb/Porin/Nucleus
pUb
Porin

Supplementary fig. 4. A POLG mutation case with α-Synuclein aggregation. (a) Example IMC image demonstrating α-Syn positive (yellow frame) and negative (white) dopaminergic (TH-positive) neurons found in the POLG02 (male, 59yrs) case. The α-Syn positive neurons showed pUb aggregates and increased signal of LonP and HTRA2. (b) Such pUb aggregates were also detected using immunofluorescence (IF) in the same case. Scale bar, 20µm

Supplementary Table. 1 Information of individual case included in the study. M-Male; F-female; MELAS- Mitochondrial encephalomyopathy, lactic acidosis, and stroke-like episodes; MERRF Myoclonic epilepsy with ragged red fibers -; CPEO- Chronic progressive external opthalmoplegia;

Case	Sex	Age	PM delay (hours)	Disease duration (years)	Case description	Lewy Pathology	Case reported in Chen et al 2021	Number of neuron analysed (Experiment 1/2)
**PD**								
PD01	Male	70	48	15	Parkinsonism without dementia	Limbic, Braak stage IV	PD02	23/35
PD02	Male	80	34	10	Parkinsonism developed with visual hallucinations	Neocortical, Braak stage VI	PD04	33/14
PD03	Male	80	88	5	Parkinsonism without dementia	Limbic, Braak stage IV	PD03	53/44
PD04	Male	81	52	10	Parkinsonism developed with visual hallucinations and confusion	Limbic, Braak stage IV	/	75/39
PD05	Male	81	13	12	Parkinsonism without dementia	Neocortical, Braak stage VI	PD05	27/40
PD06	Male	83	105	11	Parkinsonism with mild cognitive impairment	Limbic, Braak stage IV	/	20/11
PD07	Male	84	80	9	Parkinsonism without dementia	Limbic, Braak stage IV	PD08	106/126
PD08	Male	90	18	11	Parkinsonism without dementia	Limbic/Brainstem, Braak stage IV	PD09	28/24
**Mitochondrial disease (Disease controls)**								
MTDNA01	Male	52	34	unknown	MELAS syndrome, m.3243 A>G mutation	No Lewy pathology	/	81/79
MTDNA02	Male	58	66	18	MERRF syndrome, m.8344 A>G mutation	No Lewy pathology	/	22/17
MTDNA03	Female	76	78	unknown	Mitochondrial disease due to inherited mutation affecting mitochondrial respiratory chain	Neuropathological findings consistent with DLB or PDD	/	14/20

POLG01	Female	23	83	5	Mitochondrial disease presented with neurodegeneration, developed seizures and progressive ataxia. *p.Ala467Thr and p.Trp748Ser POLG1 mutation*.	No Lewy pathology	POLG02	24/19
POLG02	Male	59	67	37	Mitochondrial disease presented with CPEO, sensorimotor neuropathy; **Parkinsonism** (age 50 years) and cognitive impairment. *p.Gly848Ser and p.Ser1104Cys POLG1 mutation*	Brainstem, Braak stage III	POLG03	21/10
POLG03	Male	79	85	23	Mitochondrial disease presented with CPEO and dysphagia. *p.Thr251Ile and p.Pro587Leu and p.Ala467Thr POLG1 mutations*	Limbic/Brainstem, Braak stage III	POLG04	147/100
**Healthy controls**								
CON01	Female	18	81	/	No Parkinsonism	No Lewy pathology	Con01	83/109
CON02	Male	19	65	/	No Parkinsonism	No Lewy pathology	Con02	126/160
CON03	Male	47	29	/	No Parkinsonism	No Lewy pathology	/	13/42
CON04	Female	54	19	/	No Parkinsonism	No Lewy pathology	/	88/80
CON05	Female	59	34	/	No Parkinsonism	No Lewy pathology	/	110/152
CON06	Female	71	72	/	No Parkinsonism	No Lewy pathology	Con05	90/46
CON07	Female	71	43	/	No Parkinsonism	No Lewy pathology	Con04	97/95
CON08	Male	88	69	/	No Parkinsonism	No Lewy pathology	Con08	108/100
CON09	Male	88	23	/	No Parkinsonism	No Lewy pathology	Con07	48/91
CON10	Male	90	80	/	No Parkinsonism	No Lewy pathology	Con09	44/52
CON11	Female	93	34	/	No Parkinsonism	No Lewy pathology	Con10	35/25

Supplementary Table 2: Statistical output from the Bayesian Estimation Modelling for Fig. 5

mean	median	HDIlo	HDIup	%InROPE	casecode	target	OXPHOS deficiency	Experiment
-0.885444426	-0.885110634	-1.09669002	-0.668620244	0	CON02	NDUFA13	Complex I	Experiment 2
-0.818079595	-0.819453331	-1.189156781	-0.448880276	0.09799804	CON02	MTCO1	Complex I	Experiment 2
-0.344305079	-0.344135919	-0.541143162	-0.150797658	0.9799804	CON02	ATP5B	Complex I	Experiment 2
0.421116554	0.422833739	-0.209098241	1.035499962	8.651826963	CON02	Porin	Complex I	Experiment 2
-0.602315183	-0.602193004	-0.856722049	-0.341710535	0.09399812	CON02	TFAM	Complex I	Experiment 2
-0.230100428	-0.231209127	-0.467603256	0.000786845	11.5197696	CON02	PGC1	Complex I	Experiment 2
-0.14517068	-0.145769462	-0.367334178	0.067206566	30.25439491	CON02	LnP	Complex I	Experiment 2
-0.716866049	-0.713778461	-1.572016835	0.147079456	3.668926621	PD02	Prohibitin	Complex I	Experiment 1
0.073355316	0.073220484	-0.185122663	0.325370037	51.8249635	CON02	Htra2	Complex I	Experiment 2
-0.100076222	-0.105830558	-0.448036716	0.277599345	36.03627927	CON02	GPS2	Complex I	Experiment 2
-0.753356495	-0.750724447	-1.290222076	-0.230774419	0.74798504	PD02	DJ1	Complex I	Experiment 2
-0.011194961	-0.011473049	-0.17988605	0.155706477	78.93942121	CON02	Ser65	Complex I	Experiment 2
-2.089553902	-2.089414178	-2.586045558	-1.592481785	0.01099978	CON04	NDUFA13	Complex I	Experiment 2
-1.738770313	-1.753690985	-3.541471881	0.002346102	0.91398172	CON04	MTCO1	Complex I	Experiment 2
0.245283557	0.2432717	-0.505651342	0.995814627	17.64664707	CON04	ATP5B	Complex I	Experiment 2
0.01199601	0.008515655	-0.895058886	0.941286737	21.08057839	CON04	Porin	Complex I	Experiment 2
0.320308697	0.321787129	-1.090213757	1.784076564	11.29277414	CON04	TFAM	Complex I	Experiment 2
-0.073504471	-0.078674056	-1.568366731	1.489293389	13.11673767	CON04	PGC1	Complex I	Experiment 2
0.176446517	0.171789865	-0.582406672	0.976769018	21.14357713	CON04	LnP	Complex I	Experiment 2
-0.629442826	-0.627243644	-1.282351219	0.026429879	3.783924322	CON11	Prohibitin	Complex IV	Experiment 1
0.107073664	0.105841827	-0.654598971	0.864674705	23.80052399	CON04	Htra2	Complex I	Experiment 2
-0.048282011	-0.05769678	-0.911001686	0.82840394	22.22855543	CON04	GPS2	Complex I	Experiment 2
-0.573774068	-0.572142488	-1.062310116	-0.089140313	2.265954681	CON06	DJ1	Complex I	Experiment 2
-0.297358231	-0.296784441	-0.788941503	0.200677738	12.66174677	CON04	Ser65	Complex I	Experiment 2
-1.457189304	-1.456561525	-1.758757677	-1.16082533	0	CON05	NDUFA13	Complex I	Experiment 2
-0.923302655	-0.921942658	-1.301236622	-0.555178132	0.01099978	CON05	MTCO1	Complex I	Experiment 2

-0.317232948	-0.317289754	-0.463435765	-0.172612958	0.32099358	CON05	ATP5B	Complex I	Experiment 2
-0.134401296	-0.134745291	-0.587562748	0.311993796	29.33341333	CON05	Porin	Complex I	Experiment 2
0.17290759	0.171382644	-0.065792295	0.412793113	25.95448091	CON05	TFAM	Complex I	Experiment 2
-0.208586395	-0.208640374	-0.401743328	-0.01218428	12.42775144	CON05	PGC1	Complex I	Experiment 2
-0.142962077	-0.142930075	-0.384354937	0.093137916	32.80634387	CON05	LnP	Complex I	Experiment 2
-0.602540325	-0.06981289	-6.692031787	6.838613173	12.32375352	POLG02	Prohibitin	Complex IV	Experiment 1
-0.397959429	-0.3979207	-0.656186676	-0.132114604	1.337973241	CON05	Htra2	Complex I	Experiment 2
0.07246519	0.071781475	-0.178554281	0.324857687	50.94798104	CON05	GPS2	Complex I	Experiment 2
-0.403336346	-0.411299767	-1.291046697	0.520939949	10.63078738	PD02	DJ1	Complex IV	Experiment 2
-0.167123287	-0.167868999	-0.416847589	0.076780512	26.96246075	CON05	Ser65	Complex I	Experiment 2
-2.332110882	-2.332608086	-2.772205216	-1.888509477	0	CON06	NDUFA13	Complex I	Experiment 2
-1.673292318	-1.673686686	-2.494892744	-0.853492511	0.03399932	CON06	MTCO1	Complex I	Experiment 2
-0.480995094	-0.481150565	-0.895083151	-0.052763609	3.046939061	CON06	ATP5B	Complex I	Experiment 2
-0.540442888	-0.53715906	-1.774874962	0.6959157	8.785824284	CON06	Porin	Complex I	Experiment 2
-0.080280157	-0.078791746	-0.788914845	0.632187254	22.48955021	CON06	TFAM	Complex I	Experiment 2
-0.985504582	-0.984890088	-1.698170656	-0.299563223	0.59298814	CON06	PGC1	Complex I	Experiment 2
-0.06748552	-0.065303491	-0.671346414	0.512567534	27.33045339	CON06	LnP	Complex I	Experiment 2
-0.489437759	-0.49267376	-1.517860345	0.561354232	8.955820884	PD02	Prohibitin	Complex IV	Experiment 1
-0.228661339	-0.228387171	-1.174345428	0.705649483	15.35569289	CON06	Htra2	Complex I	Experiment 2
-0.505139325	-0.507822797	-1.542075782	0.526795601	9.263814724	CON06	GPS2	Complex I	Experiment 2
-0.37972907	-0.375969396	-1.055721491	0.279266433	11.56076878	CON06	DJ1	Complex IV	Experiment 2
-0.810959528	-0.811296285	-1.384092684	-0.245182015	0.6799864	CON06	Ser65	Complex I	Experiment 2
-1.796495084	-1.796192007	-2.062886643	-1.55018236	0	CON07	NDUFA13	Complex I	Experiment 2
-1.371906492	-1.372201076	-1.756674705	-0.983261678	0	CON07	MTCO1	Complex I	Experiment 2
-0.312213629	-0.312369323	-0.46924773	-0.159326981	0.3749925	CON07	ATP5B	Complex I	Experiment 2
0.987818634	0.987191582	0.614359445	1.364235544	0.00099998	CON07	Porin	Complex I	Experiment 2
0.265934414	0.261241407	0.003396286	0.534988875	10.15379692	CON07	TFAM	Complex I	Experiment 2
0.269773454	0.269609953	0.012923932	0.533266578	9.524809504	CON07	PGC1	Complex I	Experiment 2
0.19747378	0.197155284	0.01245931	0.384338657	14.98170037	CON07	LnP	Complex I	Experiment 2

-0.387206644	-0.391461752	-1.419583377	0.646247321	11.40677186	CON11	Prohibitin	Complex V	Experiment 1
0.396679278	0.39693533	0.150236293	0.642626206	0.93598128	CON07	Htra2	Complex I	Experiment 2
0.304709205	0.304247129	-0.010260794	0.625134661	9.335813284	CON07	GPS2	Complex I	Experiment 2
-0.321362416	-0.3221429	-0.679279253	0.043660823	8.800823984	PD01	DJ1	Complex V	Experiment 2
0.038145863	0.038425004	-0.188820682	0.271409439	58.6298274	CON07	Ser65	Complex I	Experiment 2
-1.47219713	-1.471705212	-1.695831792	-1.244995173	0	CON08	NDUFA13	Complex I	Experiment 2
-0.37269196	-0.373604536	-0.881962613	0.149512709	11.1447771	CON08	MTCO1	Complex I	Experiment 2
0.119258542	0.119421529	-0.042473675	0.27354525	40.08319834	CON08	ATP5B	Complex I	Experiment 2
0.47703058	0.477539892	0.159378218	0.787478297	0.9249815	CON08	Porin	Complex I	Experiment 2
0.114516493	0.114040537	-0.138504422	0.359712465	41.11417772	CON08	TFAM	Complex I	Experiment 2
-0.006376353	-0.006544732	-0.17720552	0.157759427	75.88048239	CON08	PGC1	Complex I	Experiment 2
0.280756435	0.280367753	0.048297257	0.509848456	6.031879362	CON08	LnP	Complex I	Experiment 2
-0.360413166	-0.359328016	-0.859367666	0.118918627	9.743805124	PD01	Prohibitin	Complex V	Experiment 1
0.142958365	0.142578648	-0.020352571	0.30698635	30.03439931	CON08	Htra2	Complex I	Experiment 2
0.058900741	0.059236467	-0.075562642	0.19290706	71.60256795	CON08	GPS2	Complex I	Experiment 2
-0.290798776	-0.287931301	-0.718870353	0.122666485	13.42473151	CON11	DJ1	Complex I	Experiment 2
0.081853504	0.081410623	-0.115851821	0.280945376	53.64692706	CON08	Ser65	Complex I	Experiment 2
-1.652534582	-1.651542454	-2.014110659	-1.30115972	0.00299994	CON09	NDUFA13	Complex I	Experiment 2
0.053544956	0.049889416	-1.088014787	1.179735638	17.59164817	CON09	MTCO1	Complex I	Experiment 2
-0.307602328	-0.307995097	-0.660232719	0.03604131	7.464850703	CON09	ATP5B	Complex I	Experiment 2
0.296985057	0.305099497	-0.415243426	0.985363528	14.07871843	CON09	Porin	Complex I	Experiment 2
0.203306238	0.201167502	-0.255003548	0.673396443	22.41755165	CON09	TFAM	Complex I	Experiment 2
-0.324766407	-0.323053993	-0.887805065	0.234327865	11.8197636	CON09	PGC1	Complex I	Experiment 2
-0.213427557	-0.21228278	-0.765803921	0.329021309	21.13157737	CON09	LnP	Complex I	Experiment 2
-0.27296622	-0.273062118	-0.414188657	-0.134506839	0.75398492	CON05	Prohibitin	Complex V	Experiment 1
-0.175972151	-0.16773343	-0.657573097	0.297810096	26.94846103	CON09	Htra2	Complex I	Experiment 2
0.43776765	0.436003531	-0.443142233	1.301580624	9.498810024	CON09	GPS2	Complex I	Experiment 2
-0.285402548	-0.285033724	-0.470966508	-0.099373946	2.444951101	MTDNA02	DJ1	Complex V	Experiment 2
-0.469996307	-0.473427814	-1.396974665	0.40179928	8.565828683	CON09	Ser65	Complex I	Experiment 2

-2.148781469	-2.148497112	-2.546129733	-1.740636939	0	CON10	NDUFA13	Complex I	Experiment 2
-0.417506144	-0.421131849	-1.656012183	0.785044091	10.29179416	CON10	MTCO1	Complex I	Experiment 2
0.303472126	0.304080406	-0.145223246	0.746138931	11.73076538	CON10	ATP5B	Complex I	Experiment 2
1.113412583	1.114238205	0.489482326	1.746892605	0.21199576	CON10	Porin	Complex I	Experiment 2
0.877066924	0.876692277	0.141806552	1.613421953	1.18597628	CON10	TFAM	Complex I	Experiment 2
0.52252389	0.517302475	0.042140704	1.04908627	2.868942621	CON10	PGC1	Complex I	Experiment 2
1.235593249	1.236066104	0.512080702	1.933346045	0.22799544	CON10	LnP	Complex I	Experiment 2
-0.227221052	-0.227118977	-0.447990421	-0.01254582	11.99276014	CON02	Prohibitin	Complex V	Experiment 1
0.72147064	0.717029016	-0.139924045	1.585510651	3.519929601	CON10	Htra2	Complex I	Experiment 2
0.689068941	0.687495015	0.296318597	1.085013703	0.36399272	CON10	GPS2	Complex I	Experiment 2
-0.276788605	-0.278296892	-0.662182107	0.127263826	13.57972841	POLG01	DJ1	Complex IV	Experiment 2
0.352272549	0.351427882	-0.217603046	0.908013446	11.45377092	CON10	Ser65	Complex I	Experiment 2
-1.365579446	-1.362356652	-2.021476104	-0.760216696	0.07699846	CON11	NDUFA13	Complex I	Experiment 2
-0.527233311	-0.520585085	-2.292830091	1.185600613	8.235835283	CON11	MTCO1	Complex I	Experiment 2
0.383367572	0.375406377	-0.617956684	1.366837779	12.00775984	CON11	ATP5B	Complex I	Experiment 2
0.362971996	0.359969576	-1.11637309	1.822480733	10.54178916	CON11	Porin	Complex I	Experiment 2
0.41395333	0.409947952	-0.523923691	1.365427695	11.02177956	CON11	TFAM	Complex I	Experiment 2
0.62692226	0.602340111	-1.08391488	2.58961652	7.571848563	CON11	PGC1	Complex I	Experiment 2
0.605698536	0.597839449	-0.986732747	2.258899321	7.611847763	CON11	LnP	Complex I	Experiment 2
-0.225104651	-0.225938557	-0.463767347	0.01726603	14.42171157	MTDNA02	Prohibitin	Complex V	Experiment 1
0.901170637	0.881048715	-0.697711391	2.598387481	4.474910502	CON11	Htra2	Complex I	Experiment 2
0.268228198	0.266042731	-1.167633558	1.687840078	11.84876302	CON11	GPS2	Complex I	Experiment 2
-0.259486312	-0.259701008	-0.442725529	-0.077449284	4.439911202	CON05	DJ1	Complex V	Experiment 2
0.041336513	0.038483072	-1.072730441	1.143993871	16.01767965	CON11	Ser65	Complex I	Experiment 2
-2.046413027	-2.045697697	-2.562371605	-1.542526946	0.00399992	MTDNA01	NDUFA13	Complex I	Experiment 2
-1.682922275	-1.688918644	-3.465621934	0.11062237	1.04197916	MTDNA01	MTCO1	Complex I	Experiment 2
0.217327682	0.215206396	-0.520362599	0.931820287	19.47361053	MTDNA01	ATP5B	Complex I	Experiment 2
-0.052015667	-0.056536763	-0.933397649	0.843294578	20.94458111	MTDNA01	Porin	Complex I	Experiment 2
0.270785605	0.270097839	-1.166528649	1.684468299	12.18775624	MTDNA01	TFAM	Complex I	Experiment 2

-0.222525419	-0.224044312	-1.595564027	1.176383889	12.72274555	MTDNA01	PGC1	Complex I	Experiment 2
0.107098076	0.10406811	-0.657592819	0.89704278	23.45353093	MTDNA01	LnP	Complex I	Experiment 2
-0.190953129	-0.190273387	-0.641296832	0.277316634	23.93252135	CON05	Prohibitin	Complex IV	Experiment 1
0.065198311	0.062324677	-0.674643808	0.84014141	25.23449531	MTDNA01	Htra2	Complex I	Experiment 2
-0.087965634	-0.088955602	-0.949997766	0.808924839	21.34657307	MTDNA01	GPS2	Complex I	Experiment 2
-0.254572236	-0.253216248	-0.461740832	-0.044721133	6.363872723	PD07	DJ1	Complex I	Experiment 2
-0.360596308	-0.36042408	-0.86174834	0.140913753	8.350832983	MTDNA01	Ser65	Complex I	Experiment 2
-1.144691034	-1.152745738	-2.589830936	0.341633802	0.80798384	MTDNA02	NDUFA13	Complex I	Experiment 2
-0.674588974	-0.700760138	-5.446918662	4.228726301	4.760904782	MTDNA02	MTCO1	Complex I	Experiment 2
0.084603962	-0.001520819	-1.043905373	1.069802892	41.3601728	MTDNA02	ATP5B	Complex I	Experiment 2
0.255674174	0.228749801	-11.40072885	12.63802232	6.950860983	MTDNA02	Porin	Complex I	Experiment 2
0.564104742	0.54831706	-0.614290673	1.712433804	3.579928401	MTDNA02	TFAM	Complex I	Experiment 2
0.246757185	0.201400918	-3.063678071	3.309236565	14.46571069	MTDNA02	PGC1	Complex I	Experiment 2
0.975627891	0.396238087	-14.28425454	15.30258749	7.974840503	MTDNA02	LnP	Complex I	Experiment 2
-0.189762159	-0.190200096	-0.50827799	0.13255174	24.29351413	CON05	Prohibitin	Complex I	Experiment 1
0.553150126	0.42776359	-3.745972684	4.758351876	8.071838563	MTDNA02	Htra2	Complex I	Experiment 2
-0.225500752	-0.308149835	-1.801135536	1.182948554	11.98876022	MTDNA02	GPS2	Complex I	Experiment 2
-0.251564019	-0.251686841	-0.392517771	-0.11424168	1.704965901	CON07	DJ1	Complex V	Experiment 2
-0.074759445	0.013663173	-2.197718919	2.349020075	23.44153117	MTDNA02	Ser65	Complex I	Experiment 2
-1.6801449	-1.678266978	-2.436185404	-0.89152037	0.0449991	MTDNA03	NDUFA13	Complex I	Experiment 2
-0.166249483	-0.164363565	-1.442427024	1.077208386	13.53172937	MTDNA03	MTCO1	Complex I	Experiment 2
0.203711746	0.205324176	-0.951071809	1.332775676	14.50070999	MTDNA03	ATP5B	Complex I	Experiment 2
1.160983927	1.165168236	-0.213609874	2.524274037	2.180956381	MTDNA03	Porin	Complex I	Experiment 2
0.273185136	0.273857611	-0.382938021	0.942529445	16.56966861	MTDNA03	TFAM	Complex I	Experiment 2
1.057285997	1.051298921	-0.615266902	2.736378522	3.603927921	MTDNA03	PGC1	Complex I	Experiment 2
0.697172574	0.698890289	-0.408921647	1.777920986	5.405891882	MTDNA03	LnP	Complex I	Experiment 2
-0.164691	-0.159529554	-11.9355533	11.9076716	5.440891182	MTDNA03	Prohibitin	Complex V	Experiment 1
0.110320081	0.11468738	-0.737190045	0.91749839	19.42961141	MTDNA03	Htra2	Complex I	Experiment 2
0.372975281	0.373922207	-1.333851513	2.094181695	9.042819144	MTDNA03	GPS2	Complex I	Experiment 2

-0.224591634	-0.215132026	-1.515406268	1.035922185	13.40773185	PD07	DJ1	Complex V	Experiment 2
-0.356740585	-0.344603938	-1.006005785	0.252217829	14.09671807	MTDNA03	Ser65	Complex I	Experiment 2
-1.233371818	-1.233861681	-1.672195967	-0.785905549	0.00299994	PD01	NDUFA13	Complex I	Experiment 2
-1.142769582	-1.144548375	-1.702583328	-0.581292412	0.08799824	PD01	MTCO1	Complex I	Experiment 2
-0.247498373	-0.247690815	-0.434790742	-0.051044912	6.293874123	PD01	ATP5B	Complex I	Experiment 2
0.289078263	0.290055692	-0.369000638	0.943927306	15.87768245	PD01	Porin	Complex I	Experiment 2
-0.255635482	-0.256621767	-0.662284983	0.145032911	16.90166197	PD01	TFAM	Complex I	Experiment 2
-0.608819374	-0.608963766	-1.028988752	-0.194898184	0.90698186	PD01	PGC1	Complex I	Experiment 2
0.079288644	0.080041541	-0.179575529	0.336606274	48.34903302	PD01	LnP	Complex I	Experiment 2
-0.142572854	-0.143858215	-0.669195575	0.393713877	27.62644747	CON09	Prohibitin	Complex I	Experiment 1
-0.087686969	-0.086962543	-0.341085059	0.164717471	47.4550509	PD01	Htra2	Complex I	Experiment 2
0.081932506	0.080164242	-0.487455629	0.662510586	27.47545049	PD01	GPS2	Complex I	Experiment 2
-0.215257359	-0.215874674	-0.668504329	0.249719184	21.05957881	CON05	DJ1	Complex IV	Experiment 2
-0.174928573	-0.172081231	-0.51061849	0.158329526	27.76644467	PD01	Ser65	Complex I	Experiment 2
-1.545641747	-1.545187568	-2.151174903	-0.94321555	0.0199996	PD02	NDUFA13	Complex I	Experiment 2
-2.088309635	-2.100354065	-3.674793471	-0.508245815	0.38799224	PD02	MTCO1	Complex I	Experiment 2
-0.720069736	-0.716244753	-1.410117744	-0.015288958	1.992960141	PD02	ATP5B	Complex I	Experiment 2
-0.79773917	-0.795145775	-1.833765933	0.206265189	3.844923102	PD02	Porin	Complex I	Experiment 2
-0.39657805	-0.394304634	-1.160785635	0.378640258	10.65678686	PD02	TFAM	Complex I	Experiment 2
-0.336292508	-0.335716151	-1.040183091	0.315263769	12.89774205	PD02	PGC1	Complex I	Experiment 2
-0.716267377	-0.712654253	-1.536188124	0.073607414	3.040939181	PD02	LnP	Complex I	Experiment 2
-0.09329051	-0.082163504	-0.819938016	0.627306133	23.19653607	CON06	Prohibitin	Complex IV	Experiment 1
-0.90021888	-0.891533216	-1.920050945	0.085268944	2.250954981	PD02	Htra2	Complex I	Experiment 2
-0.515930481	-0.518085206	-1.097521685	0.051942834	4.932901342	PD02	GPS2	Complex I	Experiment 2
-0.198086947	-0.197873079	-0.525123112	0.136542063	23.07553849	CON05	DJ1	Complex I	Experiment 2
-0.323458638	-0.322903917	-0.829801989	0.204917017	12.4947501	PD02	Ser65	Complex I	Experiment 2
-1.786738188	-1.788656681	-2.260836734	-1.326340859	0.01799964	PD03	NDUFA13	Complex I	Experiment 2
-0.955426002	-0.957258203	-3.305702212	1.434681067	4.275914482	PD03	MTCO1	Complex I	Experiment 2
-0.189760814	-0.192543218	-0.834966771	0.471846386	21.54856903	PD03	ATP5B	Complex I	Experiment 2

0.922873708	0.920613406	-0.7541283	2.616509579	3.945921082	PD03	Porin	Complex I	Experiment 2
0.396845529	0.397519511	-0.399181808	1.201631511	9.603807924	PD03	TFAM	Complex I	Experiment 2
0.411539955	0.411683672	-0.705118345	1.489208172	10.35179296	PD03	PGC1	Complex I	Experiment 2
0.37395716	0.366316907	-0.433965952	1.227286975	11.16377672	PD03	LnP	Complex I	Experiment 2
-0.060279337	-0.060156913	-0.234128684	0.118304164	63.55772885	CON07	Prohibitin	Complex V	Experiment 1
0.410742473	0.409828956	-0.595436389	1.398653704	10.38279234	PD03	Htra2	Complex I	Experiment 2
0.449295321	0.450108121	-0.53358553	1.376542519	9.180816384	PD03	GPS2	Complex I	Experiment 2
-0.172038365	0.270547578	-16.25853576	13.90013913	9.935801284	MTDNA03	DJ1	Complex V	Experiment 2
0.729989587	0.710320806	-0.304010577	1.803321906	3.654926901	PD03	Ser65	Complex I	Experiment 2
-1.47812916	-1.477446892	-1.8931087	-1.062056708	0	PD05	NDUFA13	Complex I	Experiment 2
-0.976142348	-0.975381988	-1.410181457	-0.558537115	0.01099978	PD05	MTCO1	Complex I	Experiment 2
-0.013359635	-0.012954479	-0.229293173	0.204551796	64.52770945	PD05	ATP5B	Complex I	Experiment 2
-0.181500585	-0.182027554	-0.755405106	0.375863282	22.73554529	PD05	Porin	Complex I	Experiment 2
0.288346581	0.289103712	-0.028921118	0.605399947	10.49979	PD05	TFAM	Complex I	Experiment 2
-0.11128146	-0.111946051	-0.578708356	0.349656767	30.11639767	PD05	PGC1	Complex I	Experiment 2
0.080934735	0.082051443	-0.254741909	0.42830951	39.86720266	PD05	LnP	Complex I	Experiment 2
0.063680881	0.093684927	-2.17409878	2.282632367	12.52674947	PD05	Prohibitin	Complex V	Experiment 1
-0.313988205	-0.312895656	-0.771687404	0.151713018	13.94572109	PD05	Htra2	Complex I	Experiment 2
-0.063753225	-0.064604666	-0.525017383	0.398310578	33.54732905	PD05	GPS2	Complex I	Experiment 2
-0.145419507	-0.145473708	-0.285161373	-0.006476309	25.90648187	CON07	DJ1	Complex I	Experiment 2
-0.235888358	-0.234440962	-0.588596341	0.1241946	19.03161937	PD05	Ser65	Complex I	Experiment 2
-1.402604401	-1.40245392	-1.817463099	-0.979301622	0.00099998	PD08	NDUFA13	Complex I	Experiment 2
-1.75503145	-1.75183253	-2.589199149	-0.949155316	0.01399972	PD08	MTCO1	Complex I	Experiment 2
-0.286494933	-0.287030316	-0.564600834	-0.008323743	7.981840363	PD08	ATP5B	Complex I	Experiment 2
0.263022942	0.264971604	-0.827778378	1.318547907	13.35573289	PD08	Porin	Complex I	Experiment 2
0.139762048	0.140195483	-0.353384152	0.631240544	27.32845343	PD08	TFAM	Complex I	Experiment 2
0.082777599	0.083541028	-0.383494212	0.552596852	31.84536309	PD08	PGC1	Complex I	Experiment 2
0.140154773	0.140117395	-0.184990299	0.462956224	32.93234135	PD08	LnP	Complex I	Experiment 2
0.066146206	0.066667544	-0.288053123	0.414977922	40.83818324	PD05	Prohibitin	Complex IV	Experiment 1

0.251756234	0.253829089	-0.449955258	0.952880484	17.18965621	PD08	Htra2	Complex I	Experiment 2
0.013915506	0.014573979	-0.676851041	0.694591711	23.83852323	PD08	GPS2	Complex I	Experiment 2
-0.114898766	-0.11554452	-0.278912449	0.04964377	41.85216296	CON09	DJ1	Complex IV	Experiment 2
0.082949007	0.083513471	-0.554045563	0.718760109	24.61750765	PD08	Ser65	Complex I	Experiment 2
-1.636513411	-1.63647079	-1.906714876	-1.358243583	0	PD07	NDUFA13	Complex I	Experiment 2
-0.856110211	-0.860778816	-1.30885925	-0.390415465	0.1749965	PD07	MTCO1	Complex I	Experiment 2
-0.15632238	-0.156541045	-0.453239603	0.139564857	30.22539549	PD07	ATP5B	Complex I	Experiment 2
0.632945114	0.633198942	0.185220414	1.071403123	0.95298094	PD07	Porin	Complex I	Experiment 2
0.315856768	0.312947741	-0.216305837	0.847840749	14.31671367	PD07	TFAM	Complex I	Experiment 2
-0.208781547	-0.210093442	-0.561697252	0.145139836	22.00055999	PD07	PGC1	Complex I	Experiment 2
0.152855486	0.151977919	-0.258230298	0.573711702	28.88142237	PD07	LnP	Complex I	Experiment 2
0.079401368	0.084449968	-0.588474671	0.738682414	23.75952481	CON06	Prohibitin	Complex I	Experiment 1
0.051428264	0.047092854	-0.392692891	0.510401783	34.79330413	PD07	Htra2	Complex I	Experiment 2
0.032750348	0.032769156	-0.413461102	0.46612881	35.75828483	PD07	GPS2	Complex I	Experiment 2
-0.114022233	-0.112058237	-0.572457929	0.359805213	33.39533209	CON09	DJ1	Complex I	Experiment 2
-0.068278538	-0.070254873	-0.527754755	0.389552452	32.99534009	PD07	Ser65	Complex I	Experiment 2
-1.313422472	-1.311926573	-1.572347808	-1.054488043	0	POLG03	NDUFA13	Complex I	Experiment 2
-0.302369135	-0.300804318	-0.856264769	0.250047841	15.93368133	POLG03	MTCO1	Complex I	Experiment 2
-0.029272216	-0.029348357	-0.200164019	0.147681028	71.82456351	POLG03	ATP5B	Complex I	Experiment 2
0.776589424	0.776345275	0.38501581	1.172866607	0.0449991	POLG03	Porin	Complex I	Experiment 2
0.523998063	0.523894292	0.127094045	0.916050467	1.644967101	POLG03	TFAM	Complex I	Experiment 2
0.066401951	0.066167206	-0.243669864	0.378397275	43.98312034	POLG03	PGC1	Complex I	Experiment 2
0.289509984	0.289293229	0.058167955	0.517479462	5.086898262	POLG03	LnP	Complex I	Experiment 2
0.097844648	0.421717294	-8.528992805	8.328781113	7.692846143	MTDNA02	Prohibitin	Complex I	Experiment 1
0.296415678	0.296630123	0.017384656	0.575934216	7.825843483	POLG03	Htra2	Complex I	Experiment 2
0.381026642	0.381777073	0.097487994	0.663919024	2.593948121	POLG03	GPS2	Complex I	Experiment 2
-0.1112919	-0.11095149	-0.455418284	0.229244539	36.63526729	PD05	DJ1	Complex I	Experiment 2
-0.027769313	-0.029088502	-0.373497912	0.329866027	42.07215856	POLG03	Ser65	Complex I	Experiment 2
-0.610557911	-0.61048518	-0.771687237	-0.44717216	0	CON02	NDUFA13	Complex IV	Experiment 2

-1.245433205	-1.245199481	-1.421065934	-1.072516444	0	CON02	MTCO1	Complex IV	Experiment 2
-0.250964628	-0.251040262	-0.340736323	-0.161428617	0.06399872	CON02	ATP5B	Complex IV	Experiment 2
0.813365751	0.813464659	0.601897585	1.028910495	0	CON02	Porin	Complex IV	Experiment 2
-0.542171397	-0.542233191	-0.718334926	-0.368990107	0	CON02	TFAM	Complex IV	Experiment 2
-0.008459547	-0.008436182	-0.20867771	0.188972483	67.75064499	CON02	PGC1	Complex IV	Experiment 2
-0.123232673	-0.123290692	-0.220003496	-0.02769142	31.70436591	CON02	LnP	Complex IV	Experiment 2
0.109372195	0.109006609	-0.298070667	0.517385508	32.99634007	PD03	Prohibitin	Complex V	Experiment 1
0.187902522	0.18811863	0.063475656	0.313405726	8.373832523	CON02	Htra2	Complex IV	Experiment 2
0.041304942	0.042413787	-0.15913534	0.240397846	62.97874043	CON02	GPS2	Complex IV	Experiment 2
-0.09889278	-0.098718455	-0.171326256	-0.029463937	51.39697206	CON02	DJ1	Complex IV	Experiment 2
0.01973468	0.019842992	-0.090939341	0.131736485	90.3701926	CON02	Ser65	Complex IV	Experiment 2
-1.418857023	-1.428711249	-3.23627786	0.499931512	0.94198116	CON03	NDUFA13	Complex IV	Experiment 2
-1.78582105	-1.756581916	-2.801348668	-0.632616235	0.18899622	CON03	MTCO1	Complex IV	Experiment 2
0.544653879	0.533570229	-0.916741773	1.981761798	6.625867483	CON03	ATP5B	Complex IV	Experiment 2
0.625836063	0.611435643	-0.394098759	1.599170125	3.919921602	CON03	Porin	Complex IV	Experiment 2
-0.133047455	-0.149358275	-2.147791985	1.961209068	13.32973341	CON03	TFAM	Complex IV	Experiment 2
-0.140692958	0.662681577	-2.02740815	3.241063757	5.636887262	CON03	PGC1	Complex IV	Experiment 2
0.966623244	0.964002185	-0.360886861	2.302440725	1.581968361	CON03	LnP	Complex IV	Experiment 2
0.127840807	0.12767617	-0.225619744	0.481818035	33.67632647	PD05	Prohibitin	Complex I	Experiment 1
0.559933592	0.5553507	-2.441257849	3.508160921	6.785864283	CON03	Htra2	Complex IV	Experiment 2
0.636473173	0.654595633	-1.027082241	2.20649128	5.246895062	CON03	GPS2	Complex IV	Experiment 2
-0.092147045	-0.0917171	-0.230772481	0.046137877	54.35291294	CON07	DJ1	Complex IV	Experiment 2
0.334173419	0.342878108	-2.953898051	3.64817017	7.656846863	CON03	Ser65	Complex IV	Experiment 2
-1.543668872	-1.541355643	-2.11698801	-0.94992378	0.01599968	CON04	NDUFA13	Complex IV	Experiment 2
-2.270757059	-2.26002818	-2.858346396	-1.742806114	0	CON04	MTCO1	Complex IV	Experiment 2
-0.007738892	-0.010133811	-0.368109204	0.351539087	46.44407112	CON04	ATP5B	Complex IV	Experiment 2
0.121714049	0.119256345	-0.557992672	0.785486751	23.87052259	CON04	Porin	Complex IV	Experiment 2
0.107877887	0.099862071	-0.833766818	1.078968676	18.68962621	CON04	TFAM	Complex IV	Experiment 2
-0.581387624	-0.585101559	-1.317946538	0.172884367	5.233895322	CON04	PGC1	Complex IV	Experiment 2

-0.046648532	-0.061307837	-0.538584216	0.524155939	30.97138057	CON04	LnP	Complex IV	Experiment 2
0.136416173	0.136572685	-0.45983075	0.752360388	25.69848603	PD08	Prohibitin	Complex IV	Experiment 1
-0.086846221	-0.096905017	-0.625325908	0.470737504	28.28743425	CON04	Htra2	Complex IV	Experiment 2
-0.248329308	-0.250822593	-0.709309001	0.227706109	17.70564589	CON04	GPS2	Complex IV	Experiment 2
-0.081209359	-0.079913583	-0.386982054	0.220103025	47.3350533	CON04	DJ1	Complex I	Experiment 2
-0.129053093	-0.129985049	-0.801354456	0.511013006	24.09451811	CON04	Ser65	Complex IV	Experiment 2
-1.157820629	-1.154303385	-1.765586664	-0.569337014	0.11299774	CON05	NDUFA13	Complex IV	Experiment 2
-1.339193791	-1.333813676	-1.916467574	-0.789949746	0.03099938	CON05	MTCO1	Complex IV	Experiment 2
-0.274760447	-0.275438365	-0.536042818	-0.013802835	7.305853883	CON05	ATP5B	Complex IV	Experiment 2
0.03784114	0.037575715	-0.639177122	0.721952185	26.16947661	CON05	Porin	Complex IV	Experiment 2
0.163727888	0.162327688	-0.264027752	0.609839188	27.75444491	CON05	TFAM	Complex IV	Experiment 2
-0.177740156	-0.17773429	-0.339708653	-0.01569064	15.26369473	CON05	PGC1	Complex IV	Experiment 2
-0.174711175	-0.174614114	-0.526338627	0.150293668	25.92248155	CON05	LnP	Complex IV	Experiment 2
0.137651326	-0.032766159	-1.502756569	1.421746668	44.87110258	PD07	Prohibitin	Complex V	Experiment 1
-0.343707355	-0.342633132	-0.725347441	0.038099043	7.230855383	CON05	Htra2	Complex IV	Experiment 2
0.162859799	0.163339607	-0.246806149	0.574734336	27.57644847	CON05	GPS2	Complex IV	Experiment 2
-0.077224628	-0.077523963	-0.213395871	0.060027687	63.47473051	CON02	DJ1	Complex I	Experiment 2
-0.192840447	-0.192882273	-0.528743986	0.165966552	23.17253655	CON05	Ser65	Complex IV	Experiment 2
-1.843532959	-1.843862113	-2.600066723	-1.107334016	0.01099978	CON06	NDUFA13	Complex IV	Experiment 2
-2.360329121	-2.360175776	-2.919212602	-1.795110901	0	CON06	MTCO1	Complex IV	Experiment 2
-0.574350982	-0.570523814	-1.013670597	-0.134933176	1.358972821	CON06	ATP5B	Complex IV	Experiment 2
-0.260356342	-0.253555031	-1.696669437	1.186742644	10.98878022	CON06	Porin	Complex IV	Experiment 2
-0.038311983	-0.032727246	-0.882622185	0.779385968	20.73758525	CON06	TFAM	Complex IV	Experiment 2
-0.868603322	-0.865465052	-1.607338284	-0.11092938	1.370972581	CON06	PGC1	Complex IV	Experiment 2
-0.229865275	-0.224588761	-0.8558214	0.403638483	20.00359993	CON06	LnP	Complex IV	Experiment 2
0.150106495	0.150217222	-0.019250937	0.324488285	27.98944021	CON07	Prohibitin	Complex IV	Experiment 1
-0.333287186	-0.331878197	-1.489652453	0.791154114	12.04375912	CON06	Htra2	Complex IV	Experiment 2
-0.414293287	-0.411521356	-1.546187747	0.716699533	10.9347813	CON06	GPS2	Complex IV	Experiment 2
-0.061579179	-0.061754379	-0.297109201	0.177876104	56.13687726	CON04	DJ1	Complex IV	Experiment 2

-0.740773974	-0.739031554	-1.518974071	0.015672663	2.911941761	CON06	Ser65	Complex IV	Experiment 2
-1.30385396	-1.303900497	-1.64700505	-0.955033552	0	CON07	NDUFA13	Complex IV	Experiment 2
-1.723185463	-1.722353942	-2.008809098	-1.430649305	0	CON07	MTCO1	Complex IV	Experiment 2
-0.3317107	-0.331876153	-0.496977608	-0.169214419	0.30799384	CON07	ATP5B	Complex IV	Experiment 2
0.932726479	0.933692175	0.558772639	1.295833038	0.00199996	CON07	Porin	Complex IV	Experiment 2
0.202151515	0.201786955	-0.009207536	0.409144249	16.14267715	CON07	TFAM	Complex IV	Experiment 2
0.203750511	0.203809598	-0.062395828	0.465286717	20.48059039	CON07	PGC1	Complex IV	Experiment 2
0.13663826	0.136205566	-0.046236157	0.311817973	33.88832223	CON07	LnP	Complex IV	Experiment 2
0.150942251	0.151279711	-0.270538023	0.580730452	28.71142577	PD08	Prohibitin	Complex I	Experiment 1
0.244871081	0.245354843	0.022272612	0.461058008	9.455810884	CON07	Htra2	Complex IV	Experiment 2
0.287356975	0.28738792	0.00155574	0.581449345	9.700805984	CON07	GPS2	Complex IV	Experiment 2
-0.06097467	-0.061219411	-0.176310272	0.054902859	74.51250975	POLG03	DJ1	Complex I	Experiment 2
-0.083508581	-0.083463385	-0.315751163	0.149246767	49.60700786	CON07	Ser65	Complex IV	Experiment 2
-0.641357511	-0.641608734	-1.018942847	-0.26986439	0.2749945	CON08	NDUFA13	Complex IV	Experiment 2
-1.733653494	-1.733503347	-2.02090217	-1.442161753	0	CON08	MTCO1	Complex IV	Experiment 2
0.035168852	0.035400358	-0.142057068	0.205756043	70.72358553	CON08	ATP5B	Complex IV	Experiment 2
0.723196031	0.722725217	0.442766652	1.003535052	0	CON08	Porin	Complex IV	Experiment 2
-0.098397408	-0.098743212	-0.383433033	0.182708045	41.9951601	CON08	TFAM	Complex IV	Experiment 2
-0.037573683	-0.037580762	-0.202225678	0.12765526	72.17555649	CON08	PGC1	Complex IV	Experiment 2
0.195525924	0.194887454	-0.003101005	0.393992348	16.95166097	CON08	LnP	Complex IV	Experiment 2
0.173836935	0.17368787	-1.4221554	1.704984306	15.58168837	POLG01	Prohibitin	Complex IV	Experiment 1
0.123686167	0.124047262	-0.017021636	0.263702939	36.79126417	CON08	Htra2	Complex IV	Experiment 2
-0.005658414	-0.005690919	-0.145953486	0.139116989	83.50432991	CON08	GPS2	Complex IV	Experiment 2
-0.060890747	-0.060650444	-0.381916865	0.272680361	44.56110878	PD01	DJ1	Complex I	Experiment 2
0.068388876	0.068308214	-0.152641932	0.288921042	54.31591368	CON08	Ser65	Complex IV	Experiment 2
-0.343752888	-0.345054677	-0.822078911	0.147413733	12.1047579	CON09	NDUFA13	Complex IV	Experiment 2
-1.193342191	-1.192135591	-1.402978359	-0.981773765	0	CON09	MTCO1	Complex IV	Experiment 2
0.084199933	0.083279195	-0.101638672	0.277127369	54.51390972	CON09	ATP5B	Complex IV	Experiment 2
0.424966788	0.424527723	0.101343943	0.73440795	2.074958501	CON09	Porin	Complex IV	Experiment 2

0.270959721	0.268973927	-0.036290628	0.586285695	12.72074559	CON09	TFAM	Complex IV	Experiment 2
0.212471279	0.212264344	-0.124210515	0.535878842	21.61856763	CON09	PGC1	Complex IV	Experiment 2
0.341993803	0.342212667	0.038658368	0.638550353	5.261894762	CON09	LnP	Complex IV	Experiment 2
0.177036692	0.177057267	-0.169919789	0.535060637	25.77248455	CON02	Prohibitin	Complex I	Experiment 1
0.118351546	0.115322637	-0.149374404	0.388161358	40.35219296	CON09	Htra2	Complex IV	Experiment 2
0.318410122	0.318116907	0.017420675	0.620480578	7.021859563	CON09	GPS2	Complex IV	Experiment 2
-0.058367695	-0.059376157	-0.233151816	0.114424185	65.10569789	POLG03	DJ1	Complex IV	Experiment 2
-0.028912855	-0.031462623	-0.645257443	0.58350894	25.93448131	CON09	Ser65	Complex IV	Experiment 2
-1.857290115	-1.859887237	-3.412086986	-0.249041769	0.39699206	CON10	NDUFA13	Complex IV	Experiment 2
-1.573590034	-1.576412591	-2.017237766	-1.115970872	0.05199896	CON10	MTCO1	Complex IV	Experiment 2
0.221472791	0.239950066	-0.972736001	1.403058544	15.88768225	CON10	ATP5B	Complex IV	Experiment 2
1.263275392	1.26585851	-0.181454075	2.742768292	1.00897982	CON10	Porin	Complex IV	Experiment 2
0.558335441	0.567432069	-0.691241141	1.789586613	5.636887262	CON10	TFAM	Complex IV	Experiment 2
0.275648364	0.280046947	-0.39670245	0.947534005	13.61672767	CON10	PGC1	Complex IV	Experiment 2
1.335546435	1.337596911	-0.965899988	3.490637594	1.677966441	CON10	LnP	Complex IV	Experiment 2
0.194361338	0.193662132	-0.141531184	0.528465695	23.58652827	POLG03	Prohibitin	Complex IV	Experiment 1
0.899390055	0.875226976	-1.79319472	3.455993512	4.425911482	CON10	Htra2	Complex IV	Experiment 2
0.552877083	0.562036078	-0.84793982	1.906425132	6.030879382	CON10	GPS2	Complex IV	Experiment 2
-0.057206712	-0.056471469	-0.405932085	0.265911782	48.69802604	MTDNA01	DJ1	Complex I	Experiment 2
0.26182266	0.25249099	-0.953762768	1.448889076	15.44069119	CON10	Ser65	Complex IV	Experiment 2
-0.856394605	-0.856481381	-1.535151188	-0.192603236	1.07197856	CON11	NDUFA13	Complex IV	Experiment 2
-1.513452593	-1.507883456	-2.136134304	-0.908941591	0.00099998	CON11	MTCO1	Complex IV	Experiment 2
-0.662198665	-0.661395716	-1.086628844	-0.240156037	0.50398992	CON11	ATP5B	Complex IV	Experiment 2
-0.838904412	-0.838790735	-1.643585744	-0.07333851	2.009959801	CON11	Porin	Complex IV	Experiment 2
-0.348835111	-0.343404719	-0.920139107	0.214096046	12.89474211	CON11	TFAM	Complex IV	Experiment 2
-0.736512479	-0.733241518	-1.592245062	0.11306187	3.898922022	CON11	PGC1	Complex IV	Experiment 2
-0.790914562	-0.791359669	-1.600433312	0.017011067	2.874942501	CON11	LnP	Complex IV	Experiment 2
0.208501909	0.201553536	-0.303057158	0.700689892	22.75354493	CON04	Prohibitin	Complex IV	Experiment 1
-0.551848435	-0.551070242	-1.438823109	0.3125317	8.046839063	CON11	Htra2	Complex IV	Experiment 2

-0.498668408	-0.497241116	-1.328850885	0.354553335	9.184816304	CON11	GPS2	Complex IV	Experiment 2
-0.05078714	-0.051288651	-0.359734427	0.256635518	46.69306614	PD01	DJ1	Complex IV	Experiment 2
-0.4003461	-0.402588643	-1.373074901	0.562047555	11.5297694	CON11	Ser65	Complex IV	Experiment 2
-1.452898581	-1.450479133	-1.961048449	-0.942025123	0.00899982	MTDNA01	NDUFA13	Complex IV	Experiment 2
-2.290294522	-2.278685204	-2.873215233	-1.750951499	0.00399992	MTDNA01	MTCO1	Complex IV	Experiment 2
-0.013248276	-0.015218743	-0.340182625	0.323258474	49.03901922	MTDNA01	ATP5B	Complex IV	Experiment 2
0.202785571	0.201207659	-0.424607554	0.81700548	21.18857623	MTDNA01	Porin	Complex IV	Experiment 2
0.309445435	0.306713542	-0.610447515	1.248388687	14.24871503	MTDNA01	TFAM	Complex IV	Experiment 2
-0.522007989	-0.524728392	-1.232156007	0.190450894	6.497870043	MTDNA01	PGC1	Complex IV	Experiment 2
0.019490894	0.009084563	-0.512825368	0.573515243	32.59334813	MTDNA01	LnP	Complex IV	Experiment 2
0.230272557	0.230869107	-0.060221294	0.515413866	16.31967361	PD01	Prohibitin	Complex I	Experiment 1
-0.06609368	-0.075052746	-0.5735531	0.4806084	31.05937881	MTDNA01	Htra2	Complex IV	Experiment 2
-0.088094978	-0.089760546	-0.582193136	0.416744485	31.38837223	MTDNA01	GPS2	Complex IV	Experiment 2
-0.025624435	-0.021939338	-0.147274027	0.087704367	88.77922442	CON08	DJ1	Complex I	Experiment 2
-0.119881743	-0.121658979	-0.838822658	0.626716572	22.11855763	MTDNA01	Ser65	Complex IV	Experiment 2
-0.931669482	-0.931122157	-1.436920645	-0.443459247	0.12799744	PD01	NDUFA13	Complex IV	Experiment 2
-1.34349272	-1.34344835	-1.680492637	-1.00171329	0.00099998	PD01	MTCO1	Complex IV	Experiment 2
-0.137085368	-0.137462718	-0.357528242	0.078186085	34.46431071	PD01	ATP5B	Complex IV	Experiment 2
0.582432693	0.58220385	0.085742704	1.092686337	2.428951421	PD01	Porin	Complex IV	Experiment 2
0.210490399	0.210629284	-0.180889335	0.610091836	22.10255795	PD01	TFAM	Complex IV	Experiment 2
-0.282527313	-0.283061958	-0.782054392	0.208040162	16.23367533	PD01	PGC1	Complex IV	Experiment 2
0.136561086	0.136788891	-0.091254587	0.361356994	34.82530349	PD01	LnP	Complex IV	Experiment 2
0.234500912	0.226371372	-0.189202951	0.681407956	19.86960261	MTDNA01	Prohibitin	Complex IV	Experiment 1
0.050971971	0.05185239	-0.200461435	0.302589353	53.62292754	PD01	Htra2	Complex IV	Experiment 2
0.450507244	0.449667254	-0.005201122	0.906090942	5.346893062	PD01	GPS2	Complex IV	Experiment 2
-0.021384063	-0.021821575	-0.304164834	0.269601954	54.8049039	MTDNA01	DJ1	Complex IV	Experiment 2
-0.021137991	-0.02032882	-0.373113397	0.340624993	44.80310394	PD01	Ser65	Complex IV	Experiment 2
-1.10747032	-1.127549658	-2.322024762	0.128969528	2.026959461	PD02	NDUFA13	Complex IV	Experiment 2
-2.693160959	-2.690347293	-3.716667531	-1.646850834	0.0149997	PD02	MTCO1	Complex IV	Experiment 2

-0.575401214	-0.577451383	-1.391366082	0.262190313	5.378892422	PD02	ATP5B	Complex IV	Experiment 2
-0.599485867	-0.59907884	-1.730248403	0.543034816	7.428851423	PD02	Porin	Complex IV	Experiment 2
-0.251972282	-0.249881185	-1.05438836	0.564117585	16.98966021	PD02	TFAM	Complex IV	Experiment 2
-0.462414948	-0.461277031	-1.068674686	0.115126767	6.150876982	PD02	PGC1	Complex IV	Experiment 2
-0.548950343	-0.55160625	-1.491746862	0.395134267	7.094858103	PD02	LnP	Complex IV	Experiment 2
0.251903485	0.251537258	0.038481893	0.465189334	7.860842783	CON08	Prohibitin	Complex I	Experiment 1
-0.77016526	-0.763997926	-1.891041818	0.300030236	4.227915442	PD02	Htra2	Complex IV	Experiment 2
-0.094965052	-0.096647069	-0.841474178	0.630833333	22.85254295	PD02	GPS2	Complex IV	Experiment 2
-0.010317565	-0.011134434	-0.170438435	0.154497656	78.08543829	PD07	DJ1	Complex IV	Experiment 2
-0.220136817	-0.221338647	-0.811534763	0.394945797	19.98260035	PD02	Ser65	Complex IV	Experiment 2
-1.656079068	-1.360263388	-6.696733241	4.044964684	1.782964341	PD03	NDUFA13	Complex IV	Experiment 2
-1.680524488	-2.128454734	-8.29792247	3.805032458	0.7149857	PD03	MTCO1	Complex IV	Experiment 2
-0.402495134	-0.453624858	-1.181169434	0.330827087	2.269954601	PD03	ATP5B	Complex IV	Experiment 2
0.89229943	1.616363855	-13.15238543	14.90173085	1.744965101	PD03	Porin	Complex IV	Experiment 2
0.304536369	0.207274264	-4.877052716	5.2817092	11.0397792	PD03	TFAM	Complex IV	Experiment 2
0.594659748	0.506853132	-5.227000482	6.641844904	6.391872163	PD03	PGC1	Complex IV	Experiment 2
0.239313502	0.160691519	-1.864205615	2.22657424	20.90758185	PD03	LnP	Complex IV	Experiment 2
0.255771315	0.256016075	-0.170135293	0.680912963	17.49965001	PD07	Prohibitin	Complex I	Experiment 1
0.320962763	0.425697166	-6.833854045	7.582679093	6.774864503	PD03	Htra2	Complex IV	Experiment 2
0.776589206	0.835646801	-9.982016752	11.841626	3.790924182	PD03	GPS2	Complex IV	Experiment 2
0.000451953	0.002768828	-0.116682232	0.108168623	92.58214836	CON08	DJ1	Complex IV	Experiment 2
0.282170158	0.266751099	-2.203974309	2.674009259	12.98774025	PD03	Ser65	Complex IV	Experiment 2
-1.050378389	-1.052004513	-1.577475045	-0.528154974	0.05599888	PD05	NDUFA13	Complex IV	Experiment 2
-1.105296145	-1.105495347	-1.479199203	-0.709591312	0.00099998	PD05	MTCO1	Complex IV	Experiment 2
0.002366196	0.002875208	-0.205244238	0.203862257	67.42065159	PD05	ATP5B	Complex IV	Experiment 2
-0.053352545	-0.05419133	-0.626640554	0.524223686	27.42145157	PD05	Porin	Complex IV	Experiment 2
0.267953287	0.268138196	-0.080936961	0.595244698	13.68372633	PD05	TFAM	Complex IV	Experiment 2
-0.16035615	-0.162583562	-0.65073582	0.318515406	25.82848343	PD05	PGC1	Complex IV	Experiment 2
-0.015633625	-0.01478221	-0.361855822	0.317675703	44.7901042	PD05	LnP	Complex IV	Experiment 2

0.266115941	0.265804518	0.092843901	0.438339242	2.906941861	CON07	Prohibitin	Complex I	Experiment 1
-0.347116073	-0.347295427	-0.813988824	0.108242381	11.10377792	PD05	Htra2	Complex IV	Experiment 2
-0.18330778	-0.189655467	-0.630348092	0.308793452	23.40553189	PD05	GPS2	Complex IV	Experiment 2
0.01041544	0.011445158	-0.345761169	0.359670012	43.10713786	PD05	DJ1	Complex IV	Experiment 2
-0.188354948	-0.18672739	-0.547736087	0.180847419	25.68148637	PD05	Ser65	Complex IV	Experiment 2
-1.007919385	-1.008593003	-1.351525024	-0.667147045	0.00199996	PD07	NDUFA13	Complex IV	Experiment 2
-1.317322541	-1.316766656	-1.502599641	-1.13506381	0	PD07	MTCO1	Complex IV	Experiment 2
-0.01167425	-0.012438322	-0.245256116	0.22402153	61.05777884	PD07	ATP5B	Complex IV	Experiment 2
0.652347038	0.652194352	0.331647124	0.973100425	0.08199836	PD07	Porin	Complex IV	Experiment 2
0.53213325	0.530581631	0.231013103	0.828939733	0.29399412	PD07	TFAM	Complex IV	Experiment 2
-0.0346826	-0.035131297	-0.413267993	0.353696429	39.4202116	PD07	PGC1	Complex IV	Experiment 2
0.498700023	0.498799094	0.209141604	0.779290547	0.39199216	PD07	LnP	Complex IV	Experiment 2
0.270222712	0.273092623	-0.358274655	0.895040145	16.00967981	MTDNA01	Prohibitin	Complex I	Experiment 1
0.292511851	0.290504779	-0.017788776	0.609130952	10.2247955	PD07	Htra2	Complex IV	Experiment 2
0.221662889	0.222230386	-0.136732783	0.580487116	20.62958741	PD07	GPS2	Complex IV	Experiment 2
0.072979166	0.100441559	-4.681922272	4.824271159	13.32273355	POLG02	DJ1	Complex IV	Experiment 2
-0.018063842	-0.017806961	-0.354085134	0.322768628	44.71310574	PD07	Ser65	Complex IV	Experiment 2
-1.438530516	-1.43742994	-1.928466276	-0.966976303	0.01199976	PD08	NDUFA13	Complex IV	Experiment 2
-1.909344886	-1.900064979	-3.004002001	-0.843963721	0.11299774	PD08	MTCO1	Complex IV	Experiment 2
-0.302334078	-0.302719157	-0.72882823	0.115090133	10.62078758	PD08	ATP5B	Complex IV	Experiment 2
0.281192399	0.280744277	-1.260269126	1.801906595	10.85778284	PD08	Porin	Complex IV	Experiment 2
0.294294163	0.296655449	-0.305171584	0.917838457	14.75170497	PD08	TFAM	Complex IV	Experiment 2
0.171986702	0.17219567	-0.433848393	0.786221686	23.06153877	PD08	PGC1	Complex IV	Experiment 2
0.066096199	0.0686257	-0.359526696	0.494033648	37.60624788	PD08	LnP	Complex IV	Experiment 2
0.281530255	0.281809016	0.012258459	0.550834637	8.698826023	PD01	Prohibitin	Complex IV	Experiment 1
0.174002843	0.176698522	-0.737745575	1.122621649	17.42065159	PD08	Htra2	Complex IV	Experiment 2
0.136598899	0.135045944	-0.658048965	0.909299173	20.91058179	PD08	GPS2	Complex IV	Experiment 2
0.117211707	0.117218394	-0.582661251	0.836613359	23.49853003	PD08	DJ1	Complex IV	Experiment 2
0.05762141	0.055884616	-0.768115748	0.890232477	21.53256935	PD08	Ser65	Complex IV	Experiment 2

-0.12831791	-0.121164562	-1.374101039	1.10692797	20.67058659	POLG01	NDUFA13	Complex IV	Experiment 2
-2.954799581	-2.947148412	-4.753007126	-1.195467316	0.1249975	POLG01	MTCO1	Complex IV	Experiment 2
-0.409752317	-0.411993527	-2.356115133	1.472011727	9.528809424	POLG01	ATP5B	Complex IV	Experiment 2
-0.687643041	-0.694023515	-3.1613098	1.59828058	5.769884602	POLG01	Porin	Complex IV	Experiment 2
-0.609320577	-0.617296875	-1.733518334	0.496295607	4.305913882	POLG01	TFAM	Complex IV	Experiment 2
0.037720468	-0.075148015	-1.464159318	1.335649907	19.81760365	POLG01	PGC1	Complex IV	Experiment 2
0.142836382	0.138430431	-1.540922182	1.961168989	14.83570329	POLG01	LnP	Complex IV	Experiment 2
0.283829564	0.283641666	0.078978302	0.487753133	3.832923342	CON08	Prohibitin	Complex IV	Experiment 1
-0.129574613	-0.116408299	-1.084419046	0.828290994	22.41255175	POLG01	Htra2	Complex IV	Experiment 2
-1.98812153	-1.98710231	-4.839857532	0.974682012	0.91098178	POLG01	GPS2	Complex IV	Experiment 2
0.127138181	0.130117462	-0.315900469	0.546098346	30.13539729	CON11	DJ1	Complex IV	Experiment 2
-0.068529304	-0.070140338	-1.289713021	1.046571258	21.48057039	POLG01	Ser65	Complex IV	Experiment 2
-0.491556603	0.001639298	-7.648500386	7.276554793	9.573808524	POLG02	NDUFA13	Complex IV	Experiment 2
-1.413607677	-1.498112685	-5.0896988	1.846622558	0.7699846	POLG02	MTCO1	Complex IV	Experiment 2
-0.364540042	-0.307018232	-3.395636364	2.396468249	11.49877002	POLG02	ATP5B	Complex IV	Experiment 2
0.547133092	0.638792284	-5.091759174	6.240288959	5.342893142	POLG02	Porin	Complex IV	Experiment 2
0.009827773	0.019589154	-0.783054092	0.80774476	45.27309454	POLG02	TFAM	Complex IV	Experiment 2
0.055746533	0.01648664	-1.810210496	1.963515268	22.95154097	POLG02	PGC1	Complex IV	Experiment 2
0.271627748	-0.037985606	-7.591662642	7.394148046	12.3097538	POLG02	LnP	Complex IV	Experiment 2
0.299546696	0.299171327	-0.332149538	0.939127983	14.35771285	CON04	Prohibitin	Complex I	Experiment 1
0.254164942	0.17493648	-5.903834149	6.043024217	8.865822684	POLG02	Htra2	Complex IV	Experiment 2
0.423923525	0.497702833	-2.847796453	3.465602039	7.763844723	POLG02	GPS2	Complex IV	Experiment 2
0.140263335	0.14035515	0.023874759	0.253503392	23.69552609	CON02	DJ1	Complex V	Experiment 2
0.199801901	-0.016561937	-6.028639319	6.054295718	10.98778024	POLG02	Ser65	Complex IV	Experiment 2
-0.493780136	-0.497005926	-0.812885507	-0.178553026	1.1749765	POLG03	NDUFA13	Complex IV	Experiment 2
-2.131829273	-2.132027842	-2.418635939	-1.84290351	0	POLG03	MTCO1	Complex IV	Experiment 2
-0.266080753	-0.266722602	-0.600244501	0.075766742	13.01573969	POLG03	ATP5B	Complex IV	Experiment 2
0.746033752	0.746551987	0.068417343	1.413786805	2.020959581	POLG03	Porin	Complex IV	Experiment 2
-0.103683071	-0.105569684	-0.895004034	0.694466279	20.44259115	POLG03	TFAM	Complex IV	Experiment 2

-0.146524453	-0.150813451	-0.661029339	0.379772695	25.76648467	POLG03	PGC1	Complex IV	Experiment 2
0.219326109	0.219352111	-0.220925294	0.66653921	21.01057979	POLG03	LnP	Complex IV	Experiment 2
0.320220223	0.239359871	-3.614735129	4.182893998	14.50170997	PD03	Prohibitin	Complex IV	Experiment 1
-0.177967086	-0.178502651	-0.569409265	0.214055984	26.22847543	POLG03	Htra2	Complex IV	Experiment 2
0.487979635	0.488002852	-0.094517226	1.063329693	5.960880782	POLG03	GPS2	Complex IV	Experiment 2
0.169404208	0.169482386	-0.312587546	0.654637353	25.46649067	PD08	DJ1	Complex I	Experiment 2
0.300610929	0.292454008	-0.037732396	0.632636654	8.032839343	POLG03	Ser65	Complex IV	Experiment 2
-1.041707949	-1.041453407	-1.263270308	-0.819156269	0	CON02	NDUFA13	Complex V	Experiment 2
-1.409830596	-1.408753333	-1.811431838	-1.012180434	0	CON02	MTCO1	Complex V	Experiment 2
-0.607403603	-0.607351096	-0.706504215	-0.507891351	0	CON02	ATP5B	Complex V	Experiment 2
0.173351232	0.173525196	-0.23006521	0.581909589	26.73246535	CON02	Porin	Complex V	Experiment 2
-0.722807403	-0.721616217	-1.020779834	-0.427008123	0.00199996	CON02	TFAM	Complex V	Experiment 2
-0.373277179	-0.37352741	-0.676851632	-0.066247646	3.702925941	CON02	PGC1	Complex V	Experiment 2
-0.300272225	-0.300123473	-0.474642487	-0.129144543	1.20897582	CON02	LnP	Complex V	Experiment 2
0.37597982	0.375701059	0.191823069	0.574159324	0.24799504	POLG03	Prohibitin	Complex I	Experiment 1
-0.132956269	-0.13283091	-0.332836315	0.070920371	35.90228195	CON02	Htra2	Complex V	Experiment 2
0.07354134	0.075352454	-0.237428818	0.390551575	42.35915282	CON02	GPS2	Complex V	Experiment 2
0.172134751	0.059522031	-2.281037169	2.359065261	27.93944121	MTDNA02	DJ1	Complex I	Experiment 2
-0.199354256	-0.199585125	-0.399142906	0.001550642	15.23869523	CON02	Ser65	Complex V	Experiment 2
-0.659938233	-0.660189889	-0.852913856	-0.466458964	0	CON05	NDUFA13	Complex V	Experiment 2
-0.583055453	-0.582952366	-0.727559076	-0.436989857	0	CON05	MTCO1	Complex V	Experiment 2
-0.441448283	-0.441361884	-0.50541394	-0.378331423	0	CON05	ATP5B	Complex V	Experiment 2
0.009996267	0.009800862	-0.196785907	0.219914836	65.21769565	CON05	Porin	Complex V	Experiment 2
-0.128724848	-0.128762826	-0.233030979	-0.022567213	29.41841163	CON05	TFAM	Complex V	Experiment 2
-0.0633077	-0.063441795	-0.204861771	0.078127508	68.45963081	CON05	PGC1	Complex V	Experiment 2
-0.255864812	-0.255680603	-0.367492042	-0.147028648	0.26799464	CON05	LnP	Complex V	Experiment 2
0.415953644	0.415938135	0.252138802	0.585304826	0.01399972	CON02	Prohibitin	Complex IV	Experiment 1
-0.254446176	-0.25436996	-0.403225915	-0.101042176	2.238955221	CON05	Htra2	Complex V	Experiment 2
0.07937142	0.079127218	-0.04959542	0.208762728	61.9647607	CON05	GPS2	Complex V	Experiment 2

0.194850494	0.184399061	-0.280647009	0.62154268	20.80858383	CON10	DJ1	Complex IV	Experiment 2
-0.169005514	-0.169239	-0.312243788	-0.025630933	17.41765165	CON05	Ser65	Complex V	Experiment 2
-1.089997386	-1.089627411	-1.494865376	-0.676702303	0.00199996	CON07	NDUFA13	Complex V	Experiment 2
-1.099837072	-1.101184584	-1.49358595	-0.705566418	0	CON07	MTCO1	Complex V	Experiment 2
-0.618888425	-0.619139577	-0.729188795	-0.50895967	0	CON07	ATP5B	Complex V	Experiment 2
1.135604822	1.136616	0.757779597	1.492875289	0	CON07	Porin	Complex V	Experiment 2
-0.012077765	-0.010749013	-0.239763053	0.217130549	61.14077718	CON07	TFAM	Complex V	Experiment 2
0.292192736	0.291912961	0.016793643	0.557098851	7.726845463	CON07	PGC1	Complex V	Experiment 2
-0.070038744	-0.069238637	-0.243051063	0.103410664	60.8347833	CON07	LnP	Complex V	Experiment 2
0.425450258	0.425607707	0.170960436	0.688885055	0.77298454	CON09	Prohibitin	Complex IV	Experiment 1
0.30563821	0.30735783	0.071244623	0.523789498	3.949921002	CON07	Htra2	Complex V	Experiment 2
0.192453388	0.192446855	-0.103408174	0.491450457	24.46651067	CON07	GPS2	Complex V	Experiment 2
0.220998287	0.220816326	0.030022132	0.423751631	9.191816164	CON10	DJ1	Complex I	Experiment 2
-0.095077216	-0.095043085	-0.326793501	0.136654641	46.850063	CON07	Ser65	Complex V	Experiment 2
-0.372374167	-0.371894239	-1.262830614	0.509375458	12.3897522	CON11	NDUFA13	Complex V	Experiment 2
-0.919632882	-0.920441017	-1.630191604	-0.197660886	0.92298154	CON11	MTCO1	Complex V	Experiment 2
-0.639376823	-0.638482477	-1.002938251	-0.291595172	0.23899522	CON11	ATP5B	Complex V	Experiment 2
-0.579240024	-0.580171734	-1.392667665	0.246520088	6.206875862	CON11	Porin	Complex V	Experiment 2
-0.36675092	-0.371763283	-1.09744139	0.360508314	10.49879002	CON11	TFAM	Complex V	Experiment 2
-0.315470391	-0.310660683	-1.130132272	0.463785308	14.71070579	CON11	PGC1	Complex V	Experiment 2
-0.382367246	-0.391765211	-1.774104457	1.027123955	10.16679666	CON11	LnP	Complex V	Experiment 2
0.441150605	0.426403573	-0.63625435	1.516222408	9.967800644	PD03	Prohibitin	Complex I	Experiment 1
-0.441469177	-0.444036729	-1.685160641	0.868660695	9.936801264	CON11	Htra2	Complex V	Experiment 2
-0.681098264	-0.679506446	-1.336629475	-0.046128745	2.511949761	CON11	GPS2	Complex V	Experiment 2
0.255000182	0.254041184	-0.142062412	0.675173133	14.18571629	PD03	DJ1	Complex I	Experiment 2
-0.888306799	-0.888628112	-1.762078968	0.0076746	2.092958141	CON11	Ser65	Complex V	Experiment 2
-0.911928082	-0.800846539	-7.191297718	5.909200802	4.323913522	MTDNA03	NDUFA13	Complex V	Experiment 2
-1.214261536	-1.217459189	-8.080563916	5.432838453	2.162956741	MTDNA03	MTCO1	Complex V	Experiment 2
-1.232781466	-1.228563238	-4.074188534	1.659303328	0.94798104	MTDNA03	ATP5B	Complex V	Experiment 2

0.272866444	0.014987619	-17.73513235	16.96184872	5.345893082	MTDNA03	Porin	Complex V	Experiment 2
0.077744112	0.344639458	-2.88599922	3.712400418	9.926801464	MTDNA03	TFAM	Complex V	Experiment 2
1.629102081	1.642480967	-11.14145224	14.81576601	2.038959221	MTDNA03	PGC1	Complex V	Experiment 2
2.874564907	0.060093631	-32.58364144	36.20328542	5.450890982	MTDNA03	LnP	Complex V	Experiment 2
0.507051084	0.507159292	0.210494759	0.804942722	0.43899122	PD07	Prohibitin	Complex IV	Experiment 1
-0.235346507	-0.237940702	-0.771721231	0.329753779	19.15561689	MTDNA03	Htra2	Complex V	Experiment 2
1.61780845	0.519819771	-17.69043593	20.74742112	3.798924022	MTDNA03	GPS2	Complex V	Experiment 2
0.266116881	0.265727301	-0.106366748	0.640224165	14.75470491	CON11	DJ1	Complex V	Experiment 2
0.11950483	0.13377535	-1.59103436	1.654278399	24.59250815	MTDNA03	Ser65	Complex V	Experiment 2
-0.968982906	-0.968091526	-1.323344246	-0.610325213	0.00199996	MTDNA02	NDUFA13	Complex V	Experiment 2
-0.537017489	-0.536945053	-0.770095361	-0.297552066	0.03199936	MTDNA02	MTCO1	Complex V	Experiment 2
-0.505797316	-0.505937217	-0.602727539	-0.407668319	0	MTDNA02	ATP5B	Complex V	Experiment 2
0.162756033	0.16330779	-0.195659124	0.527164862	28.70642587	MTDNA02	Porin	Complex V	Experiment 2
-0.62542664	-0.625430546	-0.980599212	-0.264778616	0.22799544	MTDNA02	TFAM	Complex V	Experiment 2
-0.692227397	-0.691108995	-1.079193477	-0.316599347	0.14599708	MTDNA02	PGC1	Complex V	Experiment 2
-0.299757136	-0.300080364	-0.540788664	-0.072292816	4.639907202	MTDNA02	LnP	Complex V	Experiment 2
0.533522029	0.526196444	-0.679974309	1.717074679	8.510829783	CON11	Prohibitin	Complex I	Experiment 1
-0.360706782	-0.360370213	-0.63647942	-0.096149253	2.799944001	MTDNA02	Htra2	Complex V	Experiment 2
-0.360774293	-0.36137168	-0.814709493	0.098508412	10.18879622	MTDNA02	GPS2	Complex V	Experiment 2
0.305851666	0.30572884	0.087021091	0.531823142	3.348933021	PD03	DJ1	Complex V	Experiment 2
-0.370596055	-0.371250002	-0.627424831	-0.122522681	1.851962961	MTDNA02	Ser65	Complex V	Experiment 2
-0.647730301	-0.646086312	-1.189652769	-0.095682754	1.788964221	PD01	NDUFA13	Complex V	Experiment 2
-0.711259731	-0.710018979	-1.280106189	-0.166346619	1.328973421	PD01	MTCO1	Complex V	Experiment 2
-0.52075917	-0.520069011	-0.665517975	-0.371099604	0.00199996	PD01	ATP5B	Complex V	Experiment 2
-0.266963692	-0.266090795	-0.924320556	0.381790568	17.05265895	PD01	Porin	Complex V	Experiment 2
-0.284742608	-0.28476422	-0.571748516	-0.000118589	8.869822604	PD01	TFAM	Complex V	Experiment 2
-0.409298649	-0.408576841	-0.976663555	0.155584042	9.031819364	PD01	PGC1	Complex V	Experiment 2
-0.274742075	-0.274586306	-0.624015722	0.065597682	12.25375492	PD01	LnP	Complex V	Experiment 2
0.733382193	0.738578572	-0.458239011	1.909995243	5.205895882	MTDNA03	Prohibitin	Complex I	Experiment 1

-0.540591401	-0.540449012	-0.996425689	-0.099158425	2.045959081	PD01	Htra2	Complex V	Experiment 2
-0.214696898	-0.213720907	-1.064604957	0.596581381	17.71964561	PD01	GPS2	Complex V	Experiment 2
0.341715968	0.334231163	-1.819797805	2.434242729	10.0797984	CON03	DJ1	Complex IV	Experiment 2
-0.793914075	-0.790873649	-1.482448979	-0.118876916	1.432971341	PD01	Ser65	Complex V	Experiment 2
-0.466662257	-0.467365412	-1.022053609	0.107133679	7.354852903	PD03	NDUFA13	Complex V	Experiment 2
-0.58304973	-0.582725348	-1.169783528	-0.022218636	3.729925401	PD03	MTCO1	Complex V	Experiment 2
-0.450959624	-0.450922296	-0.636249671	-0.264421467	0.03099938	PD03	ATP5B	Complex V	Experiment 2
1.507234373	1.508678586	0.838241627	2.152475357	0.00299994	PD03	Porin	Complex V	Experiment 2
0.073823605	0.074157835	-0.258644463	0.420235896	40.78918422	PD03	TFAM	Complex V	Experiment 2
0.662875082	0.662865115	0.302798228	1.023094887	0.16899662	PD03	PGC1	Complex V	Experiment 2
0.234415531	0.234718538	-0.116951128	0.592188741	19.07361853	PD03	LnP	Complex V	Experiment 2
0.737453334	0.735025833	-0.021789787	1.526649219	1.588968221	CON03	Prohibitin	Complex IV	Experiment 1
0.365665919	0.366444043	-0.104591599	0.822101785	10.20179596	PD03	Htra2	Complex V	Experiment 2
0.435829292	0.435835178	-0.08142443	0.948110781	7.632847343	PD03	GPS2	Complex V	Experiment 2
0.452806377	0.452776803	-0.241599251	1.143736467	9.043819124	MTDNA03	DJ1	Complex I	Experiment 2
-0.053233779	-0.060712615	-0.576094437	0.484399882	28.70942581	PD03	Ser65	Complex V	Experiment 2
-0.046678015	-0.047471271	-2.286868775	1.998396794	12.72474551	PD05	NDUFA13	Complex V	Experiment 2
-0.29193746	-0.28777371	-1.274052231	0.681737089	14.52770945	PD05	MTCO1	Complex V	Experiment 2
-0.499691791	-0.49239124	-1.081269967	0.121936612	2.833943321	PD05	ATP5B	Complex V	Experiment 2
1.271030218	1.263057832	-3.2796385	5.886401378	3.121937561	PD05	Porin	Complex V	Experiment 2
-0.129007669	-0.128973438	-1.559158064	1.349538001	18.06263875	PD05	TFAM	Complex V	Experiment 2
0.756602805	0.72256341	-4.14547605	5.337934153	4.769904602	PD05	PGC1	Complex V	Experiment 2
0.211659561	0.186027496	-2.118849727	2.563372302	11.33077338	PD05	LnP	Complex V	Experiment 2
0.978834632	1.087661943	-0.605449936	2.894764815	1.573968521	CON10	Prohibitin	Complex IV	Experiment 1
0.736733259	0.585847819	-3.917058457	5.070986171	5.372892542	PD05	Htra2	Complex V	Experiment 2
1.07254144	1.047258435	-2.906857853	5.212217541	3.883922322	PD05	GPS2	Complex V	Experiment 2
0.523161882	0.531068334	-2.53805167	3.42223908	6.839863203	PD05	DJ1	Complex V	Experiment 2
0.031894335	0.002093827	-1.924903363	2.055197249	13.36573269	PD05	Ser65	Complex V	Experiment 2
-1.085654765	-0.944883267	-6.28372399	3.844366619	2.704945901	PD07	NDUFA13	Complex V	Experiment 2

-0.574544649	-0.715005337	-6.5377001	4.991011889	4.638907222	PD07	MTCO1	Complex V	Experiment 2
-0.696238332	-0.707681916	-1.291427344	-0.123868285	0.60698786	PD07	ATP5B	Complex V	Experiment 2
0.761085957	0.548313317	-6.688515433	7.562977516	5.874882502	PD07	Porin	Complex V	Experiment 2
0.052234231	-0.182706611	-5.231097901	5.040198798	12.24675506	PD07	TFAM	Complex V	Experiment 2
-0.662461662	-0.606598014	-2.140742284	1.004401825	2.878942421	PD07	PGC1	Complex V	Experiment 2
-0.088501105	-0.097938993	-0.989535217	0.824566786	35.70428591	PD07	LnP	Complex V	Experiment 2
0.98888026	0.989821966	0.477818593	1.492856841	0.17799644	CON10	Prohibitin	Complex I	Experiment 1
-0.175555413	-0.177649831	-1.108845689	0.851088099	21.32957341	PD07	Htra2	Complex V	Experiment 2
0.293571095	-0.148931895	-3.644813588	2.951423818	18.96662067	PD07	GPS2	Complex V	Experiment 2
0.60656095	0.13613453	-4.290359835	5.615982036	16.31067379	PD03	DJ1	Complex IV	Experiment 2
0.005679612	-0.036949734	-5.192060345	5.52340748	10.67178656	PD07	Ser65	Complex V	Experiment 2

Supplementary Table 3: Statistical output from the Bayesian Estimation Modelling for Fig. 6c

mean	HDI%	95% HDI lower	95% HDI upper	%InROPE	casecode	target	Experiment
1.210475	95	0.871404	1.55111	0.001	PD03	ClpP	Experiment 1
-0.03388	95	-2.52902	2.389476	27.34045	PD03	DJ1	Experiment 2
0.315652	95	-0.4711	1.047823	13.25273	PD03	DRP1	Experiment 1
0.411976	95	-8.31308	8.299317	7.973841	PD03	GPS2	Experiment 2
0.808881	95	0.476645	1.123116	0.045999	PD03	HSP60	Experiment 1
0.431185	95	-7.8943	9.067214	4.786904	PD03	HTRA2	Experiment 2
1.087761	95	-6.41463	7.960885	4.091918	PD03	LonP	Experiment 2
0.428266	95	-0.21562	1.066081	8.437831	PD03	MFN2	Experiment 1
0.61926	95	0.24765	0.971759	0.450991	PD03	Parkin	Experiment 1
0.042157	95	-2.51065	2.576012	20.38459	PD03	PGC1	Experiment 2
0.590649	95	0.087575	1.098405	2.040959	PD03	PHB1	Experiment 1
0.32801	95	-0.09118	0.76605	9.957801	PD03	PINK1	Experiment 1
0.461954	95	-0.48271	1.377999	9.456811	PD03	Porin	Experiment 1
0.455123	95	-12.8823	14.51715	4.209916	PD03	Porin	Experiment 2
9.014166	95	-59.8399	75.7353	0.437991	PD03	Ser65	Experiment 2
0.459229	95	-0.18853	1.070461	7.225855	PD03	SIRT3	Experiment 1
0.350801	95	0.003538	0.708899	6.167877	PD03	TFAM	Experiment 1
0.171959	95	-0.5356	1.06786	16.67567	PD04	ClpP	Experiment 1
0.136955	95	-0.84709	1.290411	18.90062	PD04	DJ1	Experiment 2
0.413299	95	-1.19549	2.054447	9.52081	PD04	DRP1	Experiment 1
0.536256	95	-4.19827	5.168804	6.413872	PD04	GPS2	Experiment 2
0.288111	95	-0.10665	0.680324	7.892842	PD04	HSP60	Experiment 1
2.644446	95	-11.4058	16.10194	2.604948	PD04	HTRA2	Experiment 2
7.085238	95	-17.6698	24.14261	2.848943	PD04	LonP	Experiment 2

0.135166	95	-0.65265	0.898225	28.32943	PD04	MFN2	Experiment 1
0.11839	95	-0.40787	0.631124	33.91932	PD04	Parkin	Experiment 1
-10.9094	95	-86.751	70.74802	6.611868	PD04	PGC1	Experiment 2
0.292638	95	-0.87972	1.442841	14.18472	PD04	PHB1	Experiment 1
-0.14349	95	-0.80071	0.478103	27.47645	PD04	PINK1	Experiment 1
0.275268	95	-0.05329	0.608973	10.89778	PD04	Porin	Experiment 1
-0.28001	95	-7.41483	8.406404	7.720846	PD04	Porin	Experiment 2
0.720292	95	-20.4911	24.57578	2.226955	PD04	Ser65	Experiment 2
0.156723	95	-0.24215	0.549926	24.55051	PD04	SIRT3	Experiment 1
0.043181	95	-0.56502	0.708505	38.61923	PD04	TFAM	Experiment 1
1.025896	95	-0.13959	2.230064	1.786964	PD07	ClpP	Experiment 1
0.067535	95	-0.11818	0.261297	60.83778	PD07	DJ1	Experiment 2
0.17348	95	-1.09447	1.387959	15.71269	PD07	DRP1	Experiment 1
0.083246	95	-0.36547	0.550233	33.77432	PD07	GPS2	Experiment 2
0.498706	95	-0.48459	1.46381	7.404852	PD07	HSP60	Experiment 1
0.10561	95	-0.40594	0.612745	29.09142	PD07	HTRA2	Experiment 2
0.225606	95	-0.17764	0.637286	19.7656	PD07	LonP	Experiment 2
0.09217	95	-0.50296	0.724673	32.32135	PD07	MFN2	Experiment 1
0.227544	95	-0.29896	0.787216	19.12062	PD07	Parkin	Experiment 1
-0.43047	95	-0.97715	0.095379	7.137857	PD07	PGC1	Experiment 2
0.251578	95	-0.1623	0.721281	14.39271	PD07	PHB1	Experiment 1
0.11246	95	-0.75719	0.940807	22.91954	PD07	PINK1	Experiment 1
0.139267	95	-1.03441	1.257936	16.99466	PD07	Porin	Experiment 1
0.209417	95	-0.59278	0.990848	18.20864	PD07	Porin	Experiment 2
0.031221	95	-0.34285	0.398541	43.13614	PD07	Ser65	Experiment 2
0.252902	95	-0.2558	0.775521	15.91368	PD07	SIRT3	Experiment 1
0.279029	95	-0.55917	1.168885	16.06468	PD07	TFAM	Experiment 1
