# Supplementary material for: Parkinson’s disease neurons exhibit alterations in mitochondrial quality control proteins
Source: NPJ Parkinsons Dis. 2023 Aug 8;9:120. doi: 10.1038/s41531-023-00564-3 (PMC10409763; doi:10.1038/s41531-023-00564-3)
Supplement: Supplementary file 2 — Reporting summary [file 41531_2023_564_MOESM2_ESM.pdf]

Corresponding author(s): Chun Chen, Amy Reeve

Last updated by author(s): May 1, 2023

Reporting Summary

Nature Research wishes to improve the reproducibility of the work that we publish. This form provides structure for consistency and transparency in reporting. For further information on Nature Research policies, see our Editorial Policies and the Editorial Policy Checklist.

Statistics

For all statistical analyses, confirm that the following items are present in the figure legend, table legend, main text, or Methods section.

n/a	Confirmed	
☐	☒	The exact sample size (n) for each experimental group/condition, given as a discrete number and unit of measurement
☐	☒	A statement on whether measurements were taken from distinct samples or whether the same sample was measured repeatedly
☐	☒	The statistical test(s) used AND whether they are one- or two-sided<br>*Only common tests should be described solely by name; describe more complex techniques in the Methods section.*
☐	☒	A description of all covariates tested
☐	☒	A description of any assumptions or corrections, such as tests of normality and adjustment for multiple comparisons
☐	☒	A full description of the statistical parameters including central tendency (e.g. means) or other basic estimates (e.g. regression coefficient) AND variation (e.g. standard deviation) or associated estimates of uncertainty (e.g. confidence intervals)
☐	☒	For null hypothesis testing, the test statistic (e.g. F, t, r) with confidence intervals, effect sizes, degrees of freedom and P value noted<br>*Give P values as exact values whenever suitable.*
☒	☐	For Bayesian analysis, information on the choice of priors and Markov chain Monte Carlo settings
☒	☐	For hierarchical and complex designs, identification of the appropriate level for tests and full reporting of outcomes
☐	☒	Estimates of effect sizes (e.g. Cohen's d, Pearson's r), indicating how they were calculated

Our web collection on statistics for biologists contains articles on many of the points above.

Software and code

Policy information about availability of computer code

Data collection	Images generated from IMC were exported as tiffs (MCD viewer, Fluidigm) and converted to pseudocoloured images for visualisation using Fiji and QuPath 0.2.3
Data analysis	Statistical analysis and graph generation was performed using R 3.6.1, ggplot2, nLME and BEST package

For manuscripts utilizing custom algorithms or software that are central to the research but not yet described in published literature, software must be made available to editors and reviewers. We strongly encourage code deposition in a community repository (e.g. GitHub). See the Nature Research guidelines for submitting code & software for further information.

Data

Policy information about availability of data

All manuscripts must include a data availability statement. This statement should provide the following information, where applicable:

- Accession codes, unique identifiers, or web links for publicly available datasets
- A list of figures that have associated raw data
- A description of any restrictions on data availability

Raw datasets generated and analysed during the current study(for all figures and supplementary materials) are available in the 'Figshare' repository (doi: 10.25405/data.ncl.22005623). Data are available under the terms of the Creative Commons Attribution 4.0 International license (CC-BY 4.0). The raw datasets are available for all figures and supplementary materials.

Field-specific reporting

Please select the one below that is the best fit for your research. If you are not sure, read the appropriate sections before making your selection.

☒ Life sciences ☐ Behavioural & social sciences ☐ Ecological, evolutionary & environmental sciences

For a reference copy of the document with all sections, see nature.com/documents/nr-reporting-summary-flat.pdf

Life sciences study design

All studies must disclose on these points even when the disclosure is negative.

Sample size	The number of cases included in each group was based on the number included in previous studies using post-mortem samples. All neurons within the substantia nigra were analysed from one section from each case (Summarized in Fig 2a and Supplementary Table 1 in the manuscript). Individual neuronal number varies from case to case due to this sampling method and the presence of neurodegeneration in some cases.
Data exclusions	No data were excluded from analysis
Replication	This study aimed to use imaging mass cytometry in FFPE brain tissue and provide profiling of mitochondrial quality control protein abundance. Multiple neurons from each individual were taken and this was replicated across a number of individuals. Several of the protein targets included here have been included in further, ongoing studies allowing further replication of these experiments.
Randomization	Samples were grouped based on presence of neurodegenerative disease and the type of disease. Three groups were studied, healthy controls (with no neurological disease), cases with Parkinson's disease and cases with mitochondrial disease caused by POLG mutations or mitochondrial DNA point mutations (m.3243A>G and m.8322A>G). This grouping is appropriate given that we wanted to study the differences between these three groups to understand more about the changes specific to PD.
Blinding	Investigators were blinded to disease group during data collection and allocations only revealed when required for analysis. Case numbers were used for blinding.

Reporting for specific materials, systems and methods

We require information from authors about some types of materials, experimental systems and methods used in many studies. Here, indicate whether each material, system or method listed is relevant to your study. If you are not sure if a list item applies to your research, read the appropriate section before selecting a response.

Materials & experimental systems

n/a	Involved in the study	
☐	☒	Antibodies
☒	☐	Eukaryotic cell lines
☒	☐	Palaeontology and archaeology
☒	☐	Animals and other organisms
☒	☐	Human research participants
☒	☐	Clinical data
☒	☐	Dual use research of concern

Methods

n/a	Involved in the study	
☒	☐	ChIP-seq
☒	☐	Flow cytometry
☒	☐	MRI-based neuroimaging

Antibodies

Antibodies used

Information of antibodies are summarized in Table 1 in the manuscript:
Anti-DJ-1 (D29E5; AB_11179085) Parkinsonism-associated deglycase-7; 5933BF Cell Signaling, Lot 5933b; KO validated by the manufacture; PMID: 32051471
Anti-GPS2 G protein pathway suppressor 2;- Provided by Dr Valentina Perissi (Boston University, US)
Anti-PGC-1α (Polyclonal; AB_2268462) Peroxisome proliferator-activated receptor gamma coactivator 1-alpha; AB3243 Millipore Lot 3111137; 1KO validated by PMID: 28611589 & 31634150
Anti-SIRT3 (Polyclonal; AB_10861832) NAD-dependent protein deacetylase sirtuin-3, mitochondrial; ab86671 Abcam Lot GR291825-4; KO validated by the manufacture;
Anti-TFAM (18G102B2E11; AB_10900340) Transcription factor A, mitochondrial; ab119684 Abcam Lot GR3292863-3 1; Validated by the MitoSciences
Anti-HSP60 (24/HSP60 (RUO)/ AB_399008) 60 kDa heat shock protein, mitochondrial; 611562 BD Transduction Lot 611563; Validated using culture model (Supplementary figure 1)
Anti-PHB1 (Polyclonal;AB_823689) Prohibitin1; 2426BF Cell Signaling Lot 2426BF; KD validated by the manufacture
Anti-ClpP (Polyclonal; AB_1078538) ATP-dependent ClpP protease proteolytic subunit, mitochondrial; HPA010649 Sigma Lot C115652; KO validated by the manufacture; PMID: 23851121
Anti-HTRA2 (Polyclonal; AB_2280094) Serine protease HTRA2, mitochondrial; AF1458 R&D systems Lot IJO0917101; KO validated by

PMID: 25531304 & 19443712
Anti-LonP1 (Polyclonal; AB_1079695) Lon protease homolog, mitochondrial; HPA002192 Sigma; KO validated by the manufacture
Anti-DRP1 (D8H5; AB_11178938) Density-regulated protein; 5391BF Cell Signaling Lot 5391BF; KD validated by the manufacture; PMID: 33520735
Anti-MFN2 (6A8;AB_2142629) Mitofusin-2; ab56889 Abcam Lot GR3224958-1; KO validated by PMID: 27228353
Anti-Parkin (Polyclonal; RRID:AB_2892811) E3 ubiquitin-protein ligase; PARK2 NBP2-41287 Novus Lot 7903-1304; Validated using culture model (Supplementary figure 1)
Anti-PINK1 (Polyclonal; AB_10127658) Serine/threonine-protein kinase, mitochondrial; BC100-494 Novus Lot M; KO validated by PMID: 23256036;30258205
Anti-Phospho Ub (Polyclonal; AB_2858191) Ubiqutin, Ser65 phosphorylated; pUbSer65; ABS1513 Millipore Lot 3117322; Validated using culture model (Supplementary figure 1)
Anti-MTND4 (9E4-2D8) Complex I subunit; (mtDNA encoded); Provided by Dr Michael F. Marusich (mAbDx, Inc., Eugene, OR)
Anti-NDUFA13 (6E1BH7; AB_10863178) Complex I; ab110240 Abcam Lot GR3229012-1; Validated by the MitoSciences
Anti-SDHA (2E3GC12FB2AE2;AB_301433) Complex II; ab14715 Abcam Lot GR3229010-1; Validated by the MitoSciences
Anti-UqCRC2 (13G12AF12BB11; AB_2213640) Complex III; ab14745 Abcam Lot GR3229018-1 Validated by the MitoSciences
Anti-MTCyB (5B3-6E3) Complex III subunit (mtDNA encoded); Provided by Dr Michael F. Marusich (mAbDx, Inc., Eugene, OR)
Anti-MTCO1 (1D6E1A8;AB_2084810) Complex IV subunit (mtDNA encoded); ab14705 Abcam Lot GR229009-1 Validated by the MitoSciences
Anti-ATB5B (3D5; AB_301438) Complex V; ab14730 Abcam Lot GR3236125-2 Validated by the MitoSciences
Anti-MTATP8 (1G3-1H11-1C3) Complex V subunit (mtDNA encoded); Provided by Dr Michael F. Marusich (mAbDx, Inc., Eugene, OR)
Anti-VDAC1/Porin1 (20B12AF2; AB_443084) Voltage-dependent anion-selective channel protein 1; Mitochondrial mass marker; ab14734 Abcam Lot GR3229011-1 Validated by the MitoSciences
Anti-TH (TH-16; AB_477569) Tyrosin hydroxylase; Dopaminergic marker; SAB4200697 Sigma Lot SLBT6779
Anti-Histone H3(24HC2LC12; AB_2532490) Histone H3; Nuclear marker; 701517 Invitrogen Lot 2010233
Anti-alpha synuclein (EP1536Y; AB_869973) phospho S129; alpha synuclein marker; ab209422, Abcam Lot GR3243223-1

Validation

Describe the validation of each primary antibody for the species and application, noting any validation statements on the manufacturer's website, relevant citations, antibody profiles in online databases, or data provided in the manuscript.
